# Community recovery after a natural disaster: Core data from a survey of communities affected by the 2010 Mt. Merapi eruptions in Central Java, Indonesia

**DOI:** 10.1016/j.dib.2020.106040

**Published:** 2020-07-19

**Authors:** Jonathan A. Muir, Michael R. Cope, Leslie R. Angeningsih, Ralph B. Brown

**Affiliations:** a The Ohio State University, 238 Townshend Hall, 1885 Neil Ave Mall, Columbus, OH 43210, United States; b Brigham Young University, 2008 JFSB Provo, UT 84602, United States; c Institute of Community Development ``APMD'', Jl. Timoho 317, Yogyakarta 55225, Indonesia

**Keywords:** Community, Disaster, Environmental hazards, Recovery, Resilience, Vulnerability

## Abstract

The ``Community Recovery after a Natural Disaster: A Survey of Communities Affected by Mt. Merapi Eruptions'' data that are described herein were gathered 16 months after the 2010 Mt. Merapi volcanic eruptions in Central Java, Indonesia. Data collection was organized as a pilot effort to document victim experiences of the disaster; including disaster preparation, mitigation, and recovery. Three-stage clustered random sampling was conducted to create a sample that was representative of varying levels of destruction experienced by victims of the eruptions as well as one that included respondents who were still living in a disaster shelter, who had returned to their previous community, and who had moved on to a new community. By drawing respondents from 10 different villages or shelter communities, a total respondent sample of 400 was collected.

Specifications table

**Table:** 

**Subject**	Social Sciences (General)
**Specific subject area**	Community based disaster recovery, resilience, and vulnerability
**Type of data**	Dataset; Data Codebook; English Survey; Bahasa Indonesia Survey
**How data were acquired**	Structured survey interviews using a formal survey questionnaire
**Data format**	Raw
**Parameters for data collection**	Three-stage clustered random sampling was used to create a sample representative of varying levels of destruction experienced by disaster victims as well as one that included respondents who were still living in a disaster shelter, had returned to their previous community, or had moved on to a new community.
**Description of data collection**	Data collection was organized as a pilot effort to document victim experiences of the disaster; including disaster preparation, mitigation, and recovery. Three-stage clustered random sampling was conducted to create a sample representative of varying levels of destruction experienced by victims of the eruptions as well as one that included respondents who were still living in a disaster shelter, who had returned to their previous community, and who had moved on to a new community. By drawing respondents from 10 different villages or shelter communities, a total respondent sample of 400 was collected.
**Data source location**	City/Town/Region: **Mt. Merapi, Central Java** Country: **Indonesia**
**Data accessibility**	Repository name: **GitHub** Data identification number: **NA** Direct URL to data: https://github.com/jonathan-a-muir/Mt.Merapi-Public Steps for downloading data: Step 1: From the main GitHub page, click on green colored “Code” tab Step 2: At the bottom of the drop-down menu, select “Download Zip” Step 3: Locate and then unzip the zip file on your computer Step 4: In the data folder, select either the .csv or .sav data file for use
**Related research article**	Muir, Jonathan A., Michael R. Cope, Leslie R. Angeningsih, Jorden E. Jackson, and Ralph B. Brown. “Migration and mental health in the aftermath of disaster: evidence from Mt. Merapi, Indonesia.” *International journal of environmental research and public health* 16, no. 15 (2019): 2726. Muir, Jonathan A., Michael R. Cope, Leslie R. Angeningsih, and Jorden E. Jackson. “To move home or move on? Investigating the impact of recovery aid on migration status as a potential tool for disaster risk reduction in the aftermath of volcanic eruptions in Merapi, Indonesia.” *International Journal of Disaster Risk Reduction* (2020): 101,478.

## Value of the data

• These data document victim experiences of a disaster from a volcanic eruption that occurred in 2010 in Central Java Indonesia. The data include information concerning disaster preparation, mitigation, and recovery for 10 communities in direct geographic proximity to Mt. Merapi.
• These data should specifically benefit disaster researchers as well as policy makers and/or NGOs interested in mitigating the effects of disasters from natural hazards. More broadly, these data may be beneficial to the general scientific community.
• These data were gathered as part of a pilot effort to document the effects of the 2010 eruption. They are intended to inform further scientific inquiry and data collection in the Mt. Merapi area. It is noteworthy, that since the time of the data collection, Mt Merapi has already experienced another series of eruptions that affects local populations.
• These data document victim experiences of a disaster in a developing country. Data are generally less available concerning these types of events in developing countries and thus these data potentially provide additional insights that have yet to be analyzed from data specific to developed countries.

## 1 Data description

### 1.1 Raw data

The complete dataset includes 485 variables for 400 respondents; it is structured to follow the sections of the survey instrument described below. The publicly available data include all sections from the survey instrument except for the household member table as this includes information that would potentially make individuals and/or household identifiable.

### 1.2 Survey instruments

The survey questionnaire was originally written and organized in English and then translated into Bahasa Indonesia. Both versions are included in the supplementary materials. They contain the following topical sections: General Information, Event, Standard of Living, Access, Economics, Community, Physical and Mental Health, Household Member Table.

### 1.3 Codebook

The codebook gives detailed information concerning the original coding structure of the 485 variables included in the complete data.

## 2 Experimental design, materials, and methods

The data were collected 16 months after the 2010 volcanic eruptions from Mt. Merapi in Central Java, Indonesia [1] in an effort to document the experiences of victims of the disaster; including their experiences related to disaster preparedness, mitigation, and recovery, as well as their overall experience of the emergency. Data were collected in structured interviews using a formal survey questionnaire. The data available on GitHub are in their original raw (as entered) version. These data were used to study various determinants and health consequences of migration in the aftermath of the disaster [2,3]. See these publications, respectively, for relevant descriptions of the revisions made to the original raw data for analytic purposes.

### 2.1 Survey questionnaire

The survey questionnaire used in the data collection interview was produced in an iterative process by a team of researchers from Indonesia and the United States. It was originally written and organized in English and then translated into Bahasa Indonesia. Translation was carried out by a translation team whose members were either native speakers of Bahasa Indonesia or English while also fluent in their non-native language of either Bahasa Indonesia or English. The translation process included translation/back-translation steps to increase the questionnaire's accuracy and cultural appropriateness.

The survey questionnaire is comprised of seven topical sections: (1) General Information, (2) Event, (3) Standard of Living, (4) Access, (5) Economics, (6) Community, (7) Physical and Mental Health, and (8) Household Member Table. Additional information related to the source material(s) from which various questions in the survey questionnaire were drawn are highlighted in the data codebook. Two foundational sources for questions included in the survey questionnaire are the Indonesia Demographic and Health Survey (IDHS) [4] as well as the fourth wave of the Indonesian Family Life Survey (IFLS) [5]; additional contributing sources are indicated below where applicable.

The “General Information” section contains six questions that measure the general demographic characteristics of a respondent. Specifically, these questions—which were primarily drawn from or adapted to maintain consistency with questions in the IDHS and the IFLS [4,5]—measure a respondent's age, marital status, biological sex, ethnicity, religious preference, and level of education.

The “Event” section of the survey questionnaire contains 19 questions covering the following subtopics: (1) natural disaster experience over the last 24 months; (2) attitude towards Natural disaster; (3) governmental or nongovernmental disaster management; and (4) governmental or nongovernmental community development. These questions are consistent with those found in social science research aimed at measuring the social correlates of a disaster [6], [7], [8].

The “standard of Living” section of the survey questionnaire is comprised of 42 questions covering the following subtopics: (1) disaster related assistance, (2) housing characteristics and conditions, and (3) access to and use of various modes of technology. Again, these questions were adapted to maintain consistency with questions in either the IDHS or the IFLS [4,5] and are consistent with those found in social science research aimed at measuring the social correlates of a disaster and/or measuring household characteristics in general.

The “Economics” section of the survey questionnaire includes 24 questions covering the following subtopics: (1) economic circumstances, (2) income, (3) non-labor income (4) borrowing, and (5) household assets. Again, these questions were primarily drawn from or adapted to maintain consistency with questions in the IDHS and the IFLS [4,5].

The “community” section of the survey questionnaire is comprised of 49 questions covering the following subtopics: (1) thoughts and feelings toward their current community; (2) experience with previous community, (3) psychological sense of community, and (4) aspects of participation in their current community. These questions were drawn from well-established metrics commonly used by community scholars [9], [10], [11], [12] with prior application to countries in Southeast Asia [13] and are consistent with items recommended by disaster scholars [6], [7], [8].

The “physical and mental health” section of the survey questionnaire is comprised of 13 questions covering the following subtopics: (1) physical health, (2) metal health, and (3) quality of life. Again, some of these questions were adapted to maintain consistency with questions in either the IDHS or the IFLS [4,5]. Also included in these subsections were questions that replicated well-known items that can, for example, be used to in the calculation of The Perceived Stress Scale [14], a depression scale [15], locus of control scales [16], and a loneliness scale [17].

The final section of the survey questionnaire, “Household Member Table” is included provide a profile of the respondents’ household. Here the respondent is asked to provide the age, marital status, religion, ethnicity, and level of education for every member of their household. This table is adapted to maintain consistency with similar demographic household tables in the IDHS and the IFLS [3,4].

### 2.2 Field research team

Data were collected by research affiliates and faculty at the Institute of Community Development Research Center ``APMD'', Yogyakarta, Indonesia. All interactions between the researchers and respondents were carried out in Bahasa Indonesia. After collection, data were translated into English and entered into a database for further statistical analysis.

### 2.3 Field location

The Mt. Merapi eruptions struck five of the Regencies that encircle the mountain. Boyolali, Klaten, Magelang, and Muntilan are located within the Province of Central Java and Sleman Regency is located within the Special Region of Yogyakarta. The damages caused by the disaster varied from one region to another. After taking into consideration costs, distance, time, and that this would be a pilot project, ten villages in Sleman Regency were chosen as the data survey location.

### 2.4 Sampling procedure

Sampling was conducted to create a sample that captured varying levels of destruction experienced by disaster victims and to create a sample that included respondents who were either still living in a disaster shelter, who had returned to their previous community, or who had moved on to a new community.

To generate a sample representative of varying levels of destruction, a geographic sampling area within Sleman Regency was created by orienting the sampling area to the radius/peak of Mt. Merapi and then dividing the sampling area into different zones. These zones varied by the extent to which they were affected by the eruptions. The zone most negatively affected by the eruptions was referred to as Zone III or “Rawan Bencana III”. In this zone, the eruptions had disastrous effect. Zone II was less affected than was Zone III, and Zone I was affected the least. Across these zones, four districts were selected as the clusters for the primary sample unit: Turi, Pakem, Cangkringan, and Ngemplak.

Within these districts, several criteria were used to choose the village/shelter communities as the clusters for the secondary sample unit. First, a district was divided along its radius from north to south, and from east to west. People who were closest to the peak of Merapi in Turi District lived in Girikerto and Wonokerto villages. In Pakem District, the people closest to the volcano lived in Turgo Village. In Cangkringan, Kinahrejo was the northernmost village with closest proximity to the volcano. Finally, in Ngemplak District, Sindumartani was the village damaged most severely. The remainder of the villages selected experienced damage that ranged from moderate to slight.

### 2.5 Respondent selection

Individual households were selected to obtain a sample of those who still lived in a shelter, those who had returned to their previous communities, and those who had moved away. Household respondent selection used a similar method to the selection process used for identifying the villages and shelters. Household residences in a village/shelter were first mapped and thereafter household selection occurred by starting at a village's or shelter community's northernmost location and then randomly selecting from east to west and gradually south. This process occurred until 40 household respondents were obtained from one village, with one respondent per household (respondents were individuals that identified as head of household). By drawing respondents from 10 different villages or shelter communities, we obtained a total respondent sample of 400.

## Ethics Statement

This study met the criteria for exemption from human subjects IRB review at Brigham Young University. The authors had no financial or personal relationships with other people or organizations that could have inappropriately influenced this study. This research was funded in part by a grant from the Brigham Young University College of Family, Home, and Social Sciences as well as in part from a Brigham Young University Mentoring Environment Grant.

## Declaration of Competing Interest

Data collection was funded in part by a grant from the College of Family, Home, and Social Sciences at Brigham Young University as well as in part by a Mentoring Environment Grant from Brigham Young University. The authors declare that they had no known personal relationships or competing financial interests that have, or that could be perceived to have, influenced the data collection and preparation process. The research procedures and protocols implemented to gather the data were evaluated and approved, on 28 Feb 2012, by the Institutional Review Board for Human Subjects (IRB) at Brigham Young University. The authors further note no ongoing financial or personal relationships with people or organizations that could inappropriately influenced the maintenance of these data.

## Appendix Supplementary materials

Image, application 1

Image, application 2

Image, application 3
