# Supplementary material for: Community recovery after a natural disaster: Core data from a survey of communities affected by the 2010 Mt. Merapi eruptions in Central Java, Indonesia
Source: Data Brief. 2020 Jul 19;32:106040. doi: 10.1016/j.dib.2020.106040 (PMC7393468; doi:10.1016/j.dib.2020.106040)
Supplement: Supplementary file 2 [file mmc2.pdf]

Petunjuk-Petunjuk:

Pewawancara: ini adalah petunjuk wawancara. Tugas anda adalah memperoleh informasi yang diperlukan dengan satu cara yang sesuai dengan budaya setempat untuk konteks ini. Jangan membaca setiap pertanyaan kata demi kata, **kecuali diperintahkan**.

Di bawah ini adalah satu contoh mengenai cara anda dapat menginformasikan kepada yang diwawancarai tentang studi ini, tujuan dan kerahasiaan untuk mendapatkan suatu persetujuan lisan.

Hallo. Nama saya ____ dan saya bekerja di STPMD "APMD". Kami sedang melakukan suatu penelitian mengenai masyarakat terkait dengan kesejahteraan keluarga dan masyarakat setelah direlokasi dari wilayah yang terkena dampak erupsi Merapi. Kami sangat menghargai partisipasi Bapak/Ibu/sdr dalam penelitian ini. Saya ingin menanyakan berbagai pertanyaan tentang Bpk/Ibu/Sdr beserta keluarga, masyarakat ini dan semua kualitas hidup anggota masyarakat ini. Informasi ini akan membantu Perguruan Tinggi untuk memahami dengan lebih baik bagaimana Bpk/Ibu/Sdr dan masyarakat telah dipengaruhi karena penempatan kembali. Penelitian ini biasanya memakan waktu antara 30 sampai 40 menit untuk melengkapinya. Apapun informasi yang Bpk/Ibu/Sdr berikan akan dijaga dengan ketat kerahasiaannya. Partisipasi dalam penelitian ini adalah sukarela, dan apabila kami sampai pada pertanyaan apapun yang Bpk/Ibu/Sdr rasa tidak berkenan untuk menjawab, silahkan memberitahu kami dan kami akan melanjutkan pada pertanyaan selanjutnya. Bpk/Ibu/Sdr dapat menghentikan wawancara ini setiap saat. Namun demikian, kami berharap bahwa Bpk/Ibu/Sdr akan berpartisipasi dalam penelitian ini karena pandangan-pandangan pribadi Bpk/Ibu/Sdr yang khas adalah sangat penting bagi kami.

Saat ini, apakah Bpk/Ibu/Sdr ingin menanyakan sesuatu kepada saya terkait dengan penelitian ini?
Bolehkan saya memulai wawancara sekarang?

RESPONDEN MENYETUJUI UNTUK DIWAWANCARAI → Wawancara dimulai
RESPONDEN TIDAK MENYETUJUI DIWAWANCARAI -> Selesai

Ini adalah beberapa saran sebelum anda melakukan wawancara:

1. Kenali dan merasa nyaman dengan pertanyaan-pertanyaan dan isi jawaban-jawaban dalam penelitian ini.
2. Lakukan wawancara di tempat yang nyaman, dimana ada yang paling sedikit gangguan dan suara.
3. Jika responden tidak segera merespon, jangan menganjurkan suatu jawaban.
4. Yakinkan tentang kerahasiaan atas jawaban-jawaban bpk/ibu/sdr.

I. INFORMASI UMUM

Nama Responden: ______________________ Nama pewawancara: ___________________________
Kabupaten:___________________________ Tanggal wawancara: ___________________________
Provinsi: ____________________________ Tgl/bulan/tahun
Kota: _______________________________ Jam mulai: ____________________________________
Kecamatan: __________________________ Jam selesai: __________________________________
Desa: _______________________________
Tempat Tinggal Sekarang: Tempat Tinggal Sekarang Yang di Shelter
Pedukuhan: ____________________________ Nama Shelter: ___________________________
RT / RW: ____________________________ RT / RW Shelter: _________________________

RESPONDEN ADALAH:
1. Kepala Rumah Tangga
2. Pasangan Kepala Rumah Tangga
3. Anggota lain Rumah Tangga

Umur**:** Berapa umur Bpk/Ibu/Sdr: ________ tahun

STATUS PERKAWINAN:
1. Tidak kawin
2. Kawin
3. Berpisah
4. Cerai hidup
5. Cerai mati

JENIS KELAMIN: 1. Laki-laki
2. Perempuan

Suku Bangsa: ☐☐

AGAMA: ☐☐

TINGKAT PENDIDIKAN: Apakah Bpk/Ibu/Sdr pernah sekolah?
1. Ya
2. Tidak→ E1

Jika ya: Apakah pendidikan tertinggi yang bpk/ibu/sdr capai? ☐☐

KODE-KODE

SUKU BANGSA:
01. Jawa
02. Sunda
03. Bali
04. Batak
05. Bugis
06. Cina
07. Madura
08. Sasak
09. Minang
10. Banjar
11. Bima-Dompu
12. Makasar
13. Nias
14. Palembang
15. Sumbawa
16. Toraja
17. Betawi
18. Dayak
19. Melayu
20. Komering
21. Ambon
22. Manado
23. Aceh
25. Orang Sumatera Selatan
26. Banten
27. Cirebon
28. Gorontalo
29. Kutai
30. Lain-lain

AGAMA
01. Islam
02. Protestant
03. Katholik
04. Hindu
05. Budha
07. konfucu
08. Lain-lain

PENDIDIKAN
01. SD
02. SMP
03. SMA
04. Akademi/D1/D2/D3 (atau Sarjana Muda (D1, D2, D3)
05. Sarjana strata1
06. Pasca Sarjana S2
07. S3
09. Tidak tahu

II. KEJADIAN

Pertanyaan-pertanyaan berikut mengenai bencana alam yang dialami oleh rumah tangga ini lebih dari 24 bulan terakhir**.**

E1. TEMUKAN BENCANA-BENCANA ALAM YANG MEMENGARUHI DAERAH ASLI BPK/IBU/SDR: (LINGKARI SEMUA YANG YANG ADA)

01. .... banjir
02. Tanah longsor,
03. Aliran lumpur
04. Erupsi
05. Abu gunung api→E1A
06. Gempa bumi
07. Tsunami
08. Angin puyuh
09. Kebakaran hutan
10. kebakaran

E1A. JIKA DIPENGARUHI OLEH LETUSAN ABU GUNUNG MERAPI, APA MACAM KERUSAKAN YANG PALING MEMPENGARUHI BPK/IBU/SDR DAN YANG MEMAKSANYA UNTUK RELOKASI?

01. Awan panas
02. Letusan gunung
03. Abu
04. Aliran lahar
05. Lahar dingin
06. Pencemaran air
07. Aliran lumpur
08. Tanah longsor
09. Kebakaran hutan
10. Gangguan Kekuatan listrik
11. Banjir

E2. APAKAH ADA ANGGOTA RUMAH TANGGA YANG MENINGGAL/HILANG KARENA BENCANA TERSEBUT?

1. Tidak

2_____________ Ya. Jumlah anggota rumah tangga

a. _______ Hubungan dengan responden _________, _____________ , _____________

E3. APAKAH ANGGOTA RUMAH TANGGA MENDERITA LUKA SERIUS ATAU SAKIT KARENA BENCANA TERSEBUT?

1.Tidak

2. _____________Ya. Jumlah anggota rumah tangga

a. _______ Hubungan dengan responden _________, _____________ , _____________

E4. APA PERSEPSI BPK/IBU/SDR MENGENAI SELURUH KEHILANGAN AKIBAT BENCANA TERSEBUT?

Kerusakan ringan 1 2 3 4 5 Kerusakan total

E5.BERAPA BANYAK ASET BISNIS RUMAH TANGGA (PERTANIAN MAUPUN YANG BUKAN PERTANIAN) HILANG AKIBAT BENCANA ITU? ________________Rupiah

E5A. KIRA-KIRA BERAPA PERSEN DARI TOTAL KESELURUHAN ASET BISNIS YANG HILANG?________ %

E6. BERAPA BANYAK ASET RUMAH TANGGA YANG BUKAN BISNIS HILANG AKIBAT BENCANA ITU?

______________Rupiah

E6A. KIRA-KIRA BERAPA PERSEN DARI TOTAL KESELURUHAN ASET YANG BUKAN BISNIS YANG HILANG?

_____________%

E7. APAKAH RUMAH YANG BPK/IBU/SDR TINGGALI SEBELUM BENCANA RUSAK ATAU HANCUR?

01. Tidak rusak
02. Rusak ringan
03. Rusak berat
04. Hancur

E8. APAKAH RUMAH BPK/IBU/SDR TELAH DIPERBAIKI ATAU DIBANGUN KEMBALI?

01. Ya 02. Tidak

E9. APAKAH BPK/IBU/SDR DAPAT ATAU DIPEROBOLEHKAN UNTUK KEMBALI KE MASYARAKATNYA YANG DULU?

01. Ya 02. Tidak 03. Tidak tahu

E10. APAKAH BPK/IBU/SDR TELAH KEMBALI KE MASYARAKAT NYA SEMULA SEJAK BENCANA?

01. Ya 02. Tidak

E11. APAKAH BPK/IBU/SDR SEKARANG TINGGAL DI PEMUKIMAN SEMENTARA? (Shelter, barrak, tempat relokasi pemerintah dll.)

01. Ya→E12
02. Tidak→E14

JIKA MASIH TINGGAL DI PERUMAHAN SEMENTARA

E12. APAKAH TEMPAT TERSEBUT ADALAH:

01. Rumah milik pribadi- keluarga
02. Rumah milik pribadi- teman
03. Rumah milik pribadi- orang lain
04. Rumah peribadatan
05. Kantor/sekolah
06. Tempat perkemahan,
07. Barrak pengungsian
08. Tempat hunian sementara (Shelter)
09. Tenda
10. Ruang terbuka

E13. SUDAH BERAPA LAMA BPK/IBU/SDR TINGGAL DI PERUMAHAN SEMENTARA TERSEBUT?

______________Hari/ atau minggu/ atau bulan/atau tahun

JIKA SEKARANG TIDAK TINGGAL DI PERUMAHAN SEMENTARA

E14. APAKAH ADA ANGGOTA KELUARGA (RUMAH TANGGA) YANG SELAMA INI MENGHABISKAN WAKTU TANPA TEMPAT TINGGAL ATAU TEMPAT HUNIAN (SHELTER) SEMENTARA?

01. ___________ Ya
02. ___________ Tidak→E17
03. ___________ Tidak tahu→E17

E15. APAKAH TEMPAT TERSEBUT ADALAH:

01. Rumah milik pribadi- keluarga
02. Rumah milik pribadi- teman
03. Rumah milik pribadi- orang lain
04. Rumah peribadatan
05. Kantor/sekolah
06. Tempat perkemahan,
07. Barrak pengungsian
08. Tempat hunian semenetara (Shelter)
09. Tenda
10. Ruang terbuka

E16. SUDAH BERAPA LAMA BPK/IBU/SDR TINGGAL DI PERUMAHAN SEMENTARA TERSEBUT? (Jika ada beberapa anggota keluarga/rumah tangga dan lamanya waktu tinggal berbeda, tanyakan salah satu dari bpk/ibu/sdr yang terlama tinggal di pemukiman sementara tersebut)

______________Hari/ atau minggu/ atau bulan

Sikap terhadap Bencana Alam

Pertanyaan-pertanyaan berikut ini menanyakan tentang sikap responden terhadap bencana alam.

E17. PADA SKALA 1 SAMPAI DENGAN 5, TANYAKAN JIKA BPK/IBU/SDR SETUJU DENGAN MENYATAKAN 1 UNTUK "SANGAT SETUJU SAMPAI DENGAN 5 UNTUK "SANGAT TIDAK SETUJU."

	Sangat setuju<br>1	Agak setuju<br>2	Biasa saja<br>3	Agak tidak setuju<br>4	Sangat tidak setuju<br>5
Saya takut dengan keganasan (kemarahan) alam.	☐	☐	☐	☐	☐
Bencana alam adalah bentuk dari hukuman Allah.	☐	☐	☐	☐	☐
Bencana alam adalah bagian dari siklus hidup alam.	☐	☐	☐	☐	☐
Saya belajar hidup harmonis dengan gunung.	☐	☐	☐	☐	☐
Saya merasa mempunyai ikatan yang dalam dengan gunung Merapi.	☐	☐	☐	☐	☐
Anggota masyarakat saya dulu dapat membaca tanda-tanda alam dan memperkirakan akan adanya erupsi dengan lebih efektif dibandingkan dengan teknologi saat ini.	☐	☐	☐	☐	☐
Hidup di lereng-lereng gunung lebih baik dibandingkan dengan tinggal di tempat yang aman.	☐	☐	☐	☐	☐
Saya akan kembali ke tempat tinggal saya yang dulu apapun resikonya.	☐	☐	☐	☐	☐

Manajemen Bencana oleh Pihak Pemerintah/Lembaga Bukan Pemerintah

Pertanyaan-pertanyaan berikut ini berkaitan dengan pengalaman responden dengan metode-metode penanganan bencana.

E18.

	**Persiapan**	**Pencegahan**	**Mitigasi**	**Respon**	**Pemulihan**
Apakah pemerintah melakukan tindakan kebencanaan […] di desa bpk/ibu/sdr sebelum/sesu	01. __Ya<br>02 .__Tidak<br>03.__Tidak Tahu	01. __Ya<br>02 .__Tidak<br>03.__Tidak Tahu	01. __Ya<br>02 .__Tidak<br>03.__Tidak Tahu	01. __Ya<br>02 .___Tidak<br>03.___Tidak Tahu	01. __Ya<br>02 .__Tidak<br>03.__Tidak Tahu

dah erupsi?					
Apakah organisasi lain yang bukan pemerintah melakukan tindakan kebencanaan […] di desa bpk/ibu/sdr sebelum/sesudah erupsi?	01. __Ya<br>02 .__Tidak<br>03.__Tidak Tahu	01. __Ya<br>02 __Tidak<br>03.___Tidak Tahu	01. __Ya<br>02 .__Tidak<br>03.___Tidak Tahu	01. __Ya<br>02 .__Tidak<br>03.___Tidak Tahu	01. __Ya<br>02 .__Tidak<br>03.___Tidak Tahu
Jika ya, apakah responden berpartisipasi dalam kegiatan /menerima bantuan tersebut?	01. __Ya<br>02 .__Tidak<br>03.__Tidak Tahu	01. __Ya<br>02 .__Tidak<br>03.__Tidak Tahu	01. __Ya<br>02 .__Tidak<br>03.__Tidak Tahu	01. __Ya<br>02 .__Tidak<br>03.__Tidak Tahu	01. __Ya<br>02 .___Tidak<br>03.__ Tidak Tahu
Jika ya, pada skala 1 sampai dengan 5, bagaimana bpk/ibu/sdr menilai efektifitas dari metode tersebut?	01. __Sangat efektif<br>02. _di atas rata-rata<br>03. _Sedang<br>04. __di bawah rata-rata<br>05. _Sangat tidak efektif	01. _Sangat efektif<br>02. __di atas rata-rata<br>03. ___Sedang<br>04. _di bawah rata-rata<br>05. _Sangat tidak efektif	01. __Sangat efektif<br>02. _di atas rata-rata<br>03. _Sedang<br>04. __di bawah rata-rata<br>05. _Sangat tidak efektif	01. __Sangat efektif<br>02. __di atas rata-rata<br>03. __Sedang<br>04. __di bawah rata-rata<br>05. __Sangat tidak efektif	01. __Sangat efektif<br>02. __di atas rata-rata<br>03. __Sedang<br>04. __di bawah rata-rata<br>05. __Sangat tidak efektif
Jika ya, apakah bpk/ibu/sdr merasa beberapa metode dibuat sama seperti tradisi budaya dan nilai bpk/ibu/sdr?	01. ___Ya<br>02 .__Tidak<br>03.__Tidak Tahu	01. ____Ya<br>02 .___ Tidak<br>03.___ Tidak Tahu	01. __Ya<br>02 .__Tidak<br>03.___Tidak Tahu	01. __Ya<br>02 .__ Tidak<br>03.___ Tidak Tahu	01. __Ya<br>02 .__ Tidak<br>03.__ Tidak Tahu

<u>Pembangunan Masyarakat oleh Pemerintah/Bukan Pemerintah</u>

Pertanyaan-pertanyaan berikut ini berkaitan dengan pengalaman responden dengan metode pembangunan masyarakat.

E19. PADA SKALA 1 SAMPAI DENGAN 5, DIMANA SKALA 1 MENUNJUKKAN "LUAR BIASA HEBAT" SAMPAI DENGAN 5 "SANGATLAH BURUK", SILAHKAN BPK/IBU/SDR MENILAI SECARA KESELURUHAN KUALITAS IMPLEMENTASI PEMBANGUNAN MASYARAKAT YANG DILAKUKAN OLEH PEMERINTAH.

	Luar biasa hebat	Di atas rata-rata	sedang	Di bawah rata-rata	Sangatlah buruk
Pembangunan					
Perencanaan					
Bangunan					
Penempatan					
Kelangsungan					

II. STANDAR HIDUP

Bagian-bagian ini berkaitan dengan standar hidup responden sejak penempatan kembali.

Bantuan

Pertanyaan-pertanyaan berikut berhubungan dengan bantuan yang diterima oleh responden dan/atau anggota keluarga/rumahtangga berkaitan dengan bencana alam (abaikan bantuan yang dihubungkan dengan bantuan kemiskinan secara umum).

SL1. APAKAH BPK/IBU/SDR ATAU APAKAH BPK/IBU/SDR SEKARANG MENERIMA BANTUAN **KEUANGAN** DARI PEMERINTAH DAN/ATAU ORGANISASI NON PEMERINTAH (ABAIKAN KELUARGA DAN TEMAN).

01. Ya, akhir-akhir ini

02. Ya, tetapi tidak lagi menerima

03. Tidak→sl2

SL1A. JIKA YA, DARI SIAPA BANTUAN ITU BERASAL:

01. Pemerintah Pusat
02. Pemerintah Daerah
03. Kelompok
04. keagamaan
05. Organisasi- organisasi Politik
06. LSM lokal
07 Lembaga Bantuan Swasta
08 Perusahaan/korporasi
09 Pemerintah
10 Asing/LSM/Lembaga donor
11 Lain-lain

SL1B. BERAPA JUMLAH TOTAL BANTUAN YANG DITERIMA BERKAITAN DENGAN BENCANA TERSEBUT? Rp.

SL1C.BERAPA PERSEN DARI TOTAL PENDAPATAN PER BULAN JUMLAH BANTUAN TERSEBUT? %

SL1D. PADA SKALA 1 SAMPAI DENGAN 5, SKALA 1 MENUNJUKKAN "SANGAT CUKUP" DAN SKALA 5 MENUNJUKKAN "SAMA SEKALI TIDAK CUKUP", APAKAH BPK/IBU/SDR MERASA JUMLAH YANG DIBERIKAN ADALAH CUKUP UNTUK KEBUTUHAN-KEBUTUHAN BPK/IBU/SDR?

Sangat cukup	1	2	3	4	5	Sama sekali tidak mencukupi

SL2. APAKAH BPK/IBU/SDR SAAT INI MENERIMA PERAWATAN DAN BANTUAN KESEHATAN DARI PEMERINTAH ATAU DARI LEMBAGA NON PEMERINTAH (ABAIKAN KELUARGA DAN TEMAN)?

01. Ya, akhir-akhir ini
02. Ya, tetapi tidak lagi menerima
03. Tidak, →SL3

SL2A. APAKAH BANTUAN TERSEBUT MENCAKUP SEMUA PERAWATAN YANG MEMADAI UNTUK LUKA-LUKA AKIBAT BENCANA ATAU LUKA-LUKA YANG DIALAMI OLEH ANGGOTA RUMAH TANGGA?

01. Ya, mencakup seluruh perawatan

02. Ya, tetapi hanya sebagian
03. Tidak, luka-luka yang khusus tidak tercakup

SL2B. SEBERAPA DEKAT FASILITAS PERAWATAN KESEHATAN YANG MENAWARKAN PERAWATAN YANG MEMADAI (UNTUK RESPONDEN ATAU ANGGOTA RUMAH TANGGA)____________Km

SL2C. PADA SKALA 1 SAMPAI DENGAN 5, SKALA 1 MENUNJUKKAN "SANGAT LUAR BIASA BAIK" DAN SKALA 5 MENUNJUKKAN "SANGAT BURUK SEKALI", SILAHKAN BPK/IBU/SDR MENILAI KUALITAS KESELURUHAN PERAWATAN YANG DITERIMA OLEH RESPONDEN DAN ANGGOTA RUMAH TANGGANYA.

Luar biasa baik 1 2 3 4 5 Sangat buruk sekali

SL3. APAKAH BPK/IBU/SDR SAAT INI MENERIMA **BANTUAN MAKANAN** DARI PEMERINTAH DAN/ATAU ORGANISASI NON PEMERINTAH (ABAIKAN KELUARGA DAN TEMAN-TEMAN).

01. Ya, akhir-akhir ini
02. Ya, tetapi sekarang tidak lagi
03. Tidak →sl4

SL3A. JIKA YA, DARI SIAPA?

01. Pemerintah Pusat
02. Pemerintah Daerah
03. Kelompok keagamaan
04. Organisasi-organisasi Politik
05. LSM lokal
06 Lembaga Bantuan swasta
07 Perusahaan/korporasi
08 Pemerintah Asing/LSM/
09 Lembaga donor lain
10 Lain-lain

SL3B. PADA SKALA 1SAMPAI DENGAN 5, SKALA 1 MENUNJUKKAN "SANGAT MEMADAI ATAU MENCUKUPI" DAN SKALA 5 MENUNJUKKAN "TIDAK MEMADAI ATAU MENCUKUPI SAMA SEKALI", APAKAH BPK/IBU/SDR MERASA BAHAN MAKANAN YANG DISEDIAKAN OLEH ORGANISASI-ORGANISASI ADALAH CUKUP MEMADAI UNTUK KEBUTUHAN MAKAN BPK/IBU/SDR SEHARI-HARI*?*

Sangat memadai atau mencukupi 1 2 3 4 5 Tidak memadai atau mencukupi sama sekali

SL3C. APAKAH DARI BEBERAPA KELOMPOK MAKANAN BERIKUT INI TIDAK DISEDIAKAN DALAM PROGRAM BANTUAN INI TETAPI ITU SANGAT PENTING ATAU POKOK UNTUK KEBUTUHAN MAKAN ?

01. karbohidrat: beras, ketela, ubi, kentang, dsb.
02. protein: ikan, ayam, daging, babi, tahu, kacang-kacangan, tempe dsb.
03. buah-buahan: pisang, mangga, nangka, tomat dsb.
04. sayur-sayuran:timun, terong, tauge, bawang merah, kol dsb.
05. susu
06. santan
07. teh
08. bumbu-bumbu dapur

SL4. APAKAH BPK/IBU/SDR SAAT INI MENERIMA UANG UNTUK MEMBAYAR SEWA TEMPAT TINGGAL DARI PEMERINTAH DAN/ATAU ORGANISASI NON PEMERINTAH (ABAIKAN KELUARGA DAN TEMAN-TEMAN).

01. Ya, akhir-akhir ini menerima
02. Ya, tetapi sekarang tidak lagi menerima, kapan menerima terakhir kali?
03. Tidak→sl5

SL4A. JIKA YA, DARI SIAPA?

01. Pemerintah Pusat
02. Pemerintah Daerah
03. Kelopok-kelompok agama
04. Organisasi-organisasi politik
05. Lembaga Swadaya Masyarakat Lokal
06. Bantuan swasta
07. Perusahaan/korporasi
08. Pemerintah Asing/LSM/Donatur

SL4B. PADA SKALA 1 SAMPAI DENGAN 5, SKALA 1 MENUNJUKKAN "SANGAT MEMAAI ATAU MENCUKUPI" DAN SKALA 5 "SANGAT TIDAK MEMADAI ATAU MENCUKUPI SAMA SEKALI", APAKAH BPK/IBU/SDR MERASA BANTUAN YANG DIBERIKAN MENCUKUPI UNTUK KEBUTUHAN-KEBUTUHAN BPK/IBU/SDR?

Sangat memadai atau mencukupi	1	2	3	4	5	Sangat tidak memadai atau mencukupi sama sekali

<u>Kharakteristik dan kondisi perumahan</u>

Pertanyaan-pertanyaan berikut ini berkaitan dengan kharakteristik dan kondisi dari tempat tinggal bpk/ibu/sdr saat ini.

SL5. STATUS KEPEMILIKAN TEMPAT TINGGAL HUNIAN SAAT INI:

01. hak milik
02. kontrak/sewa
03. milik pemerintah
04. milik orangtua/keluarga/saudara
05. lain-lain

SL6. TIPE TEMPAT TINGGAL HUNIAN

01. satu unit satu lantai
02. satu unit dengan beberapa lantai
03. berbagi tembok duplek satu lantai
04. berbagai tembok duplek dengan beberapa lantai
05. beberapa unit satu lantai
06. beberapa unit beberapa lantai
07. rumah dengan tiang (model panggung)
08. rumah tingkat/ bangunan apartemen
09. unit-unit rumah pertokoan
10. lain-lain

SL7. KONDISI SANITASI SECARA UMUM:

	Ya	Tidak
Rumah dikelilingi oleh kotoran manusia dan hewan (tidak termasuk buangan kotoran secara terbuka).		
Rumah dikelilingi oleh tumpukan sampah.		
Rumah dikelilingi oleh genangan air.		
Ada kumpulan hewan di bawah/atau disamping rumah		
Rumah memiliki cukup aliran udara/ventilasi.		
Halaman terpelihara dengan baik dan bersih.		
Rumah memiliki dapur di luar		
Ruang masak dan ruang tidur ada di ruangan yang sama		

SL8. PERKIRAKAN UKURAN RUMAH DALAM METER PERSEGI ☐.☐☐☐ meter persegi

SL9. PERKIRAKAN BERAPA BANYAK RUANG (RUANG TIDUR, RUANG KELUARGA, DAPUR, KAMAR MANDI DSB) YANG ADA DALAM RUMAH BPK/IBU/SDR ☐☐ Ruangan

SL10. APA SUMBER UTAMA AIR MINUM BAGI RUMAHTANGGA RESPONDEN

01. PIPA AIR
02. SUMUR/POMPA (LISTRIK, TANGAN)
03. AIR SUMUR
04. SUMBER MATA AIR,
05. AIR HUJAN
06. SUNGAI/AIR PANCURAN
07. KOLAM/KOLAM IKAN
08. KOLAM TAMPUNGAN AIR
09. BELI
10. LAIN-LAIN

SL11. APAKAH BPK/IBU/SDR MELAKUKAN SESUATU UNTUK MEMBUAT AIR AMAN UNTUK DIMINUM?

01. Merebus
02. Menambahkan pemutih/klorine
03. Menyaring air (keramik, pasir, komposit dsb)
04. Menghilangkan bakteri dengan sinar matahari
05. Membiarkan mengendap
06. Tidak melakukan apa-apa
07. Lain-lain
08. Tidak tahu

SL12. BERAPA HARI DALAM SATU MINGGU BPK/IBU/SDR MEMPUNYAI AKSES TERHADAP AIR TERSEBUT (lingkari satu jenis saja)

01. Setiap hari
02. Setiap 3 hari
03. Dua kali seminggu
04. Sekali seminggu
05. Sangat jarang

SL13. DIMANA LETAK SUMBER AIR MINUM TERSEBUT?

01. Di dalam rumah
02. Di Luar rumah

SL14A. JIKA DI LUAR RUMAH, BERAPA LAMA UNTUK MENGAMBIL DARI SUMBER, KEMUDIAN MENGUMPULKAN AIR DAN KEMBALI KE RUMAH? Menit

SL14B. JIKA DI LUAR RUMAH, BERAPA JAUH DARI SUMBER KE RUMAH? Km

SL15. APAKAH AIR DIGUNAKAN UNTUK KEPERLUAN LAIN, SEPERTI MANDI DAN MENCUCI, JUGA DIAMBIL DARI SUMBER YANG SAMA DENGAN UNTUK AIR MINUM?

01. Ya
02. Tidak

SL16. APA SUMBER UTAMA AIR YANG DIGUNAKAN OLEH RUMAH TANGGA BPK/IBU/SDR UNTUK KEPERLUAN LAIN SEPERTI MEMASAK, MANDI, CUCI DAN/ATAU MENCUCI TANGAN?

01. Air pipa
02. Sumur/pompa (listrik, tangan)
03. Air sumur
04. Sumber mata air
05. Air hujan
06. Sungai/air pancuran
07. Kolam/kolam ikan
08. Tampungan air
09. Beli
10. Lain-lain

SL17. BERAPA HARI DALAM SATU MINGGU BPK/IBU/SDR MEMPUNYAI AKSES TERHADAP AIR TERSEBUT (lingkari satu jenis saja)

01. Setiap hari
02. Setiap tiga hari
03. Dua kali seminggu
04. Sekali seminggu
05. Jarang sekali

SL18. DIMANA SUMBER AIR UTAMA YANG DIGUNAKAN UNTUK KEBUTUHAN RUMAHTANGGA SEPERTI MASAK, MANDI, CUCI, DAN ATAUA/ MENCUCI TANGAN TERLETAK?

1. di dalam rumah
2. di luar rumah

SL18A. JIKA DI LUAR RUMAH, BERAPA LAMA BPK/IBU/SDR MENGAMBIL DARI SUMBER, KEMUDIAN MENGUMPULKAN AIR DAN KEMBALI KE RUMAH? Menit

SL18B. JIKA DI LUAR RUMAH, BERAPA JAUH DARI SUMBER AIR KE RUMAH? Km

SL19. DIMANA PEMILIK RUMAH KEBANYAKAN MENGGUNAKAN TOILET KAMAR KECIL

1. Milik pribadi dengan septik tank
2. Milik pribadi tanpa septik tank

Toilet umum/berbarengan

3. Sungai kecil/ Sungai/Selokan (tanpa toilet)
4. Halaman/lapangan/semak-semak/hutan (tanpa toilet)
5. Buangan kotoran
6. Kolam/kolam ikan
7. Kandang hewan
8. Sungai/jeram/sungai kecil
9. Lain-lain

SL20. DIMANA RUMAHTANGGA INI MENGOSONGKAN ATAU MEMBUANG KOTORANNYA?

01. Selokan pembuangan (mengalir)
02. Selokan pembuangan (berhenti)
03. Lubang permanen
04. Dibuang ke sungai
05. Kolam/kolam ikan/danau/kubangan
06. Lubang (tanpa garis permanen)
07. Sawah atau kebun lain
08. Laut atau pantai
09. Lain-lain

SL21. APAKAH BPK/IBU/SDR MENERIMA BANTUAN AIR (AKSES KE ATAU SANITASI)DARI PEMERINTAH DAN/ATAU ORGANISASI NON PEMERINTAH (ABAIKAN KELUARGA DAN TEMAN-TEMAN)

1. Ya,akhir-akhir ini
2. Ya, tetapi sekarang tidak lagi
3. Tidak→

SL21A. JIKA YA, UNTUK APA?(lingkari semua yang memenuhi)

01. Air minum
02. Kebutuhan rumah tangga
03. Sanitasi (tetes klorine, saringan air/filter)
04. Toilet
05. Saluran/pipa buangan kotoran ,

06 lain-lain, biarkan bpk/ibu/sdr menjelaskannya ______________________

SL21B. JIKA YA, DARI SIAPA?

01. Pemerintah Pusat
02. Pemerintah Daerah
03. Kelompok-kelompok agama
04. Organisasi-organisasi politik
05. LSM local

06 Bantuan swasta

06. Perusahaan/korporasi-korporasi
07. Pemerintah asing/LSM/Donatur
08. Lain-lain __________

SL21C. SILAHKAN BPK/IBU/SDR MENILAI SECARA KESELURUHAN KUALITAS BANTUAN DARI SKALA 1 SAMPAI DENGAN SKALA 5, SKALA 1 MENUNJUKKAN "SANGAT BAIK" DAN SKALA 5 MENUNJUKKAN "SANGAT BURUK."

Sangat baik 1 2 3 4 5 Sangat buruk

SL22. BAGAIMANA RUMAH TANGGA INI MEMBUANG SAMPAHNYA?

01. Dibuang di keranjang sampah, dikumpulkan oleh petugas kebersihan
02. Layanan sanitasi/kebersihan
03. Dibakar
04. Dibuang ke sungai/sungai kecil/atau tempat buangan kotoran
05. Dibuang di halaman dan dibiarkan membusuk
06. Dibuang di kubangan
07. Di hutan, gunung

08. Laut, danau, pantai
09. Sawah atau kebun lain
10. Lain-lain

SL23. APAKAH BPK/IBU/SDR MENYIMPAN MAKANAN YANG MUDAH RUSAK DI DALAM KULKAS?
01. Ya
02. Tidak
03. Tidak punya kulkas

SL24. MACAM BAHAN BAKAR/KOMPOR YANG DIGUNAKAN UNTUK MEMASAK?
01. Listrik
02. Gas
03. Kompor minyak tanah
04. Kayu bakar
05. Arang
06. Tidak masak
07. Lain-lain

SL27. SILAHKAN BPK/IBU/SDR MENILAI SELURUH KONDISI HIDUP BPK/IBU/SDR DARI SKALA 1 SAMPAI DENGAN SKALA 5, SKALA 1 MENUNJUKKAN "SANGAT BAIK" DAN SKALA 5 MENUNJUKKAN "SANGAT BURUK."

Sangat baik 1 2 3 4 5 Sangat buruk

<u>Akses</u>

Pertanyaan-pertaanyaan berikut ini menanyakan tentang akses bpk/ibu/sdr terhadap berbagai macam teknologi dan penggunaannya.

SL26. APAKAH RUMAH TANGGA INI MENGGUNAKAN LISTRIK?
01. Ya
02. Tidak

SL27. APA SUMBER LISTRIKNYA?
01. Matahari
02. Jaringan lisrik (pemerintah/perusahaan listrik)

SL28. APAKAH RUMAH TANGGA INI MEMILIKI TELEVISION?
01. Ya
02. Tidak

SL29. APAKAH RUMAH TANGGA INI MEMILIKI KOMPUTER?
01. Ya
02. TIDAK

SL30. APAKAH BPK/IBU/SDR PERNAH MENGGUNAKAN INTERNET SEBELUMNYA?
01. Ya
02. Tidak →SL33

SL30A. DIMANA BPK/IBU/SDR MENGGUNAKAN INTERNET? (lingkari semua yang memenuhi)
01. Di rumah
02. Di persewaan computer/internet
03. Di sekolah

SL30B. APA YANG DILAKUKAN DENGAN INTERNET? (lingkari yang memenuhi)
01. Ngobrol dengan teman dan keluarga
02. Hiburan
03. Mengerjakan PR
04. Mencari informasi
05. Mengirim email
06. Berita
07. _______Lain-lain

SL31. APAKAH RUMAH TANGGA INI MEMPUNYAI JARINGAN TELPON (JARINGAN TETAP RUMAH)?

01. Ya | 02. Tidak→SL35

SL31. SEBERAPA SERING BPK/IBU/SDR MENGGUNAKAN TELPON DALAM AKHIR BULAN LALU

01	02	03	04	05
Tidak pernah	1-15 kali	6-10 kali	11-15 kali	16 kali atau lebih

SL32. APAKAH RESPONDEN MENGGUNAKAN HP, APAKAH ITU MILIK SENDIRI, TEMAN ATAU KELUARGA?

01. Tidak →SL42
02. Ya, HP pribadi
03. Ya, HP milik orang lain

SL33.APA YANG BPK/IBU/SDR LAKUKAN DENGAN MENGGUNAKAN HP TERSEBUT?

01. Pekerjaan sekarang
02. Mencari pekerjaan
03. Mengorganisir rapat, pertemuan-pertemuan dan janjian
04. Upaya melegakan relaks
05. Bicara dengan orang-orang lain di desa
06. Bicara dengan orang-orang yang saat ini tidak tinggal di desa
07. Musik, games atau hiburan
08. Lain-lain
    Silahkan bpk/ibu/sdr menjelaskan sendiri ______

SL34. APAKAH BPK/IBU/SDR MENGGUNAKAN HP LEBIH UNTUK BEKERJA ATAU BERKOMUNIKASI DENGAN TEMAN-TEMAN DAN KELUARGA?

01. Bekerja
02. Teman-teman dan keluarga
03. Lain-lain

SL35. SEBERAPA SERING BPK/IBU/SDR MEMBELI PULSA UNTUK MELAKUKAN TELPON?

01. Tidak pernah
02. Sangat jarang
03. Kadang-kadang
04. Hampir selalu

SL36. DIMANA BPK/IBU/SDR MEMBELI PULSA UNTUK TELPON?

01. Di toko di desa saya
02. Di toko di desa lain
03. Lain-lain
    Silahkan bpk/ibu/sdr menjelaskan sendiri ______________________________

SL37. JUMLAH PANGGILAN TELPON YANG BPK/IBU/SDR TERIMA DALAM BULAN LALU MEMALUI HP BPK/IBU/SDR________________________JUMLAH PANGGILAN TELPON

SL38. SEJAUH MANA JARAK DESA ATAU KOTA TERDEKAT? _________________Meter

SL39. BERAPA KALI BPK/IBU/SDR MELAKUKAN PERJALANAN KE DESA ATAU KOTA TERDEKAT DALAM SEBULAN? ________________________KALI

SL40. SEJAUHMANA JARAK RUMAH BPK/IBU/SDR DARI PASAR TERDEKAT? ________________Km

SL41. BERAPA BANYAK PASAR YANG TERSEDIA DALAM JARAK 5 KM DIMANA BPK/IBU/SDR TINGGAL?

_______________________________ JUMLAH PASAR

SL42. JENIS ALAT TRANSPORTASI UTAMA:

01. Jalan kaki
02. Mobil/ truk
03. Sepeda motor
04. Bis
05. Sepeda

III. EKONOMI

EC1. APAKAH BPK/IBU/SDR SEKARANG SEDANG BEKERJA?
01. SEDANG BEKERJA
02. TIDAK SEDANG BEKERJA ⇓
EC1A. JIKA BPK/IBU/SDR SEKARANG TIDAK SEDANG BEKERJA, SUDAH BERAPA LAMA BPK/IBU/SDR MENJADI PENGANGGURAN?___________________________ BULAN→ EC23

Pewawancara: jika bpk/ibu/sdr sekarang tidak sedang bekerja, silahkan bpk/ibu/sdr menjawab pertanyaan-pertanyaan berikut berdasarkan pada pekerjaan bpk/ibu/sdr yang paling akhir dalam 12 bulan yang lalu. Jika bpk/ibu/sdr tidak bekerja dalam 12 bulan terakhir →

EC2. BEBERAPA ORANG MELAKUKAN PEKERJAAN YANG MENDAPAT PEMBAYARAN TUNAI ATAU BARANG ATAU TIDAK DIBAYAR. YANG LAIN MENJUAL BARANG, MEMPUNYAI USAHA KECIL ATAU BEKERJA PADA KELUARGA DI LAHAN PERTANIAN KELUARGA ATAU DITEMPAT USAHA KELUARGA, APAKAH BPK/IBU/SDR MEMPUNYAI PEKERJAAN YANG IA SECARA TERUS-MENERUS SEDIKITNYA SATU JAM PADA MINGGU YANG LALU?
01. Ya
02. Tidak

EC3. APA JENIS PEKERJAAN BPK/IBU/SDR (KEGIATAN PEKERJAAN YANG BPK/IBU/SDR LAKUKAN UMUMNYA TERLIBAT DI DALAMNYA)?
01. Profesional, Teknis
02. Manajemen dan administrasi
03. Juru tulis, pegawai kantoran
04. Penjual
05. Jasa layanan
06. Bekerja dipertanian
07. Buruh pabrik, pekerja produksi
08. _________lain-lain (sebutkan secara khusus)
09. Tidak tahu

EC4. UMUMNYA, APAKAH BPK/IBU/SDR BEKERJA DI PERTANIAN ATAU DI BISNIS YANG BUKAN PERTANIAN?
01. Pertanian→EC5
02. Bukan pertanian→EC18

Pasangan

EC5. JIKA RESPONDEN MENIKAH, APAKAH PASANGAN BPK/IBU/SDR SEKARANG BEKERJA?
01. YA
02. TIDAK →EC

EC6. JIKA PASANGAN BPK/IBU/SDR SEKARANG TIDAK BEKERJA, APAKAH IA PERNAH BEKERJA DALAM 12 BULAN TERAKHIR?
01. YA
02. TIDAK

EC7. JIKA YA, APA PEKERJAANNYA (KEGIATAN/KERJA UTAMA YANG BPK/IBU/SDR LAKUKAN)?
10. Profesional, Teknis
11. Manajemen dan administrasi
12. Juru tulis, pegawai kantoran
13. Penjual
14. Jasa layanan
15. Bekerja dipertanian
16. Buruh pabrik, pekerja produksi
17. _________lain-lain (sebutkan secara khusus)
18. Tidak tahu

EC8. BERAPA KILOMETER IA MELAKUKAN PERJALANAN KE TEMPAT KERJA (SEKALI JALAN)?
______________Km

EC9. SECARA KESELURUHAN APAKAH MERASA PUAS DENGAN PEKERJAANNYA SEKARANG?

Sangat puas sekali 1 2 3 4 5 Sangat tidak puas

Pendapatan

Pertanyaan-pertanyaan berikut ini berkaitan dengan pendapatan bpk/ibu/sdr dan pendapatan yang bukan dari tenaga kerja

EC10. PENDAPATAN BULANAN RUMAH TANGGA BPK/IBU/SDR DARI SUMBER-SUMBER RUMAH TANGGA SECARA INTERNAL?
(PEKERJAAN, KEKAYAAN, BISNIS, PEKERJAAN PASANGAN, PEKERJAAN ANAK-ANAK)
Rp _____________

EC11. APAKAH PENDAPATAN BPK/IBU/SDR TETAP/DAPAT DIPASTIKAN DARI BULAN KE BULAN [APAKAH BPK/IBU/SDR TAHU BERAPA BANYAK BPK/IBU/SDR AKAN MEMPEROLEH TIAP-TIAP BULANNYA]?
01. Ya 02. Tidak

EC12. FAKTOR-FAKTOR APA YANG MENYUMBANGKAN KEPASTIAN PENDAPATAN TERSEBUT?
_____________________________ Silahkan dijelaskan sendiri

EC13. SIAPA YANG MEMBERIKAN PENDAPATAN PALING PASTI DALAM KELUARGA TERSEBUT?
01. Bapak kepala rumah tangga
02. Ibu kepala rumah tangga
03. Anak laki-laki dewasa
04. Anak perempuan dewasa
05. Anak laki-laki termuda
06. Anak perempuan termuda
07. Lain-lain_________________________

EC14. SIAPA YANG MENYUMBANGKAN UANG PALING BANYAK KEPADA KEUANGAN KELUARGA?
01. Bapak kepala rumah tangga
02. Ibu kepala rumah tangga
03. Anak laki-laki dewasa
04. Anak perempuan dewasa
05. Anak laki-laki termuda
06. Anak perempuan termuda
07. Lain-lain ________________________

EC15. APAKAH RUMAH TANGGA MENERIMA SEJUMLAH UANG DARI ORANG YANG TIDAK TINGGAL BERSAMA DI RUMAH BPK/IBU/SDR?
01. Ya 02. Tidak
JIKA YA:
EC33A. APA HUBUNGAN KELUARGA DENGAN RUMAH TANGGA INI ___________________________________
EC33B. BERAPA BANYAK BPK/IBU/SDR MENGIRM SETIAP BULANNYA? Rp._________

Pendapatan bukan dari kerja

EC16. JUMLAH PENDAPATAN RUMAH TANGGA TIAP BULANNYA YANG BERASAL DARI SUMBER LUAR (PEMERINTAH, LSM, ARISAN, KELUARGA DAN TEMAN-TEMAN)?

01. < RP 500.000
02. RP 501.000 – RP 1000.000
03. RP 1001.000 – RP 2.000.000
04. RP2001.000 – RP 2.500.000
05. RP2501.000 – RP 3.000.000
06. RP 3001.000 – RP 3500.000,-
07. RP.3501.000 – RP 4.000.000,-
08. RP 4001.000 >

EC17. PELENGKAP LAIN, PENDAPATAN BUKAN BERUPA UANG

	jumlah	Perkiraan jumlah	Persentase dari	Sumber

		dalam Rp.	jumlah pendapatan	
**Makanan**				
**Perabot rumah tangga**				
**Peralatan pertanian**				
**Hewan ternak**				

Sumber:

01. Pemerintah Pusat
02. Pemerintah Daerah
03. Kelompok-kelompok agama
04. Organisasi-organisasi politik
05. LSM lokal
06. Donatur swasta
07. Perusahaan/korporasi-korporasi
08. Pemerintah asing/LSM/donatur
09. lain-lain __________

Pinjaman

Sekarang kita akan menanyakan tentang pinjaman atau hutang dari bukan keluarga atau teman-teman dalam 12 bulan terakhir

EC18. APAKAH BPK/IBU/SDR ATAU BEBERAPA ANGGOTA RUMAH TANGGA MENCOBA MEMINJAM SEJUMLAH UANG ATAU BARANG DARI SUMBER SELAIN DARI KELUARGA ATAU TEMAN-TEMAN BPK/IBU/SDR DALAM 12 BULAN TERAKHIR INI?

01. Ya
02. Tidak

EC19. UNTUK TUJUAN APA PINJAMAN TERBESAR SELAMA 12 BULAN TERAKHIR INI?

01. Kelahiran
02. Kematian
03. Pernikahan
04. Pembayar akad
05. Upacara sosial
06. Untuk membeli barang-barang rumah tangga
07. Pengobatan
08. Pendidikan
09. Renovasi rumah
10. Untuk membeli rumah
11. Untuk membeli kebutuhan pertanian (bibit, pestisida)
12. Untuk membeli /memperbaiki peralatan pertanian
13. Untuk membeli tanah
14. Untuk membeli ternak
15. Untuk membeli kebutuhan unggas
16. Bisnis perikanan
17. Untuk membeli/ memperbaiki (kendaraan komersiil roda tiga)
18. Untuk membeli/ memperbaiki kapal
19. Untuk membeli/ memperbaiki jala ikan
20. Bahan-bahan untuk industri penginapan
21. Modal untuk bisnis lain
22. Pengeluaran harian
23. Arisan
24. Untuk membantu anggota rumah tangga, keluarga atau teman
25. Untuk membeli/ memperbaiki kendaraan
26. Membayar hutang
27. Transport/perjalanan
28. Lain-lain

Harta benda kepemilikan kekayaan (asset) rumah tangga

Selanjutnya, kami ingin mengetahui harta kekayaan (asset) yang dimiliki oleh yang diwawancarai dan anggota yang lain dari rumah tangganya.

EC20. APAKAH YANG DIWAWANCARAI MEMILIKI ASSET BERIKUT?

01. Ya
02. Tidak→EC51

	Jenis (lingkari yang ada)	Berapa banyak […] dalam jumlah	Apakah bpk/ibu/sdr mempunyai kepemilikan atas itu semua […]	Jika tidak, apa yang tersedia sesungguhnya […]?	Apakah […] tersedia karena bencana?
**Ternak**	A B C D E F G	________	1. YA<br>2. TIDAK	□□	1. YA<br>2. TIDAK
**Kendaraan**	A B C D E F G H	________	1. YA<br>2. TIDAK	□□	1. YA<br>2. TIDAK
**Peralatan rumah tangga**	A B C D E F G H I J	________	1. YA<br>2. TIDAK	□□	1. YA<br>2. TIDAK
**Perkakas kecil**	A B C D E F G	________	1. YA<br>2. TIDAK	□□	1. YA<br>2. TIDAK
**Perhiasan**	A B C D E F	________	1. YA<br>2. TIDAK	□□	1. YA<br>2. TIDAK
**Perabotan rumah tangga**	A B C D E F	________	1. YA<br>2. TIDAK	□□	1. YA<br>2. TIDAK

Kode-kode

Ternak

A. Sapi/Sapi perah/kerbau
B. Kambing/domba
C. Kuda/keledai atau bagal
D. Babi
E. Unggas
F. Kolam ikan
G. Lain-lain

Kendaraan

A. Mobil/truk
B. Kapal motor
C. Kapal dayung
D. Sepeda
E. Sepeda motor
F. Traktor
G. Kendaraan air
H. Gerobak ditarik hewan

Peralatan rumah tangga

A. Radio
B. Tape rekaman
C. TV
D. Kulkas
E. Mesin jahit
F. VCD
G. HP
H. Charger Batery
I. Telpon
J. Telpon genggam

Perkakas kecil

A. Gergaji
B. Kapak
C. Palu
D. Garpu
E. Bajak
F. Cangkul
G. Penggaruk

Perhiasan

A. Cincin
B. Jam tangan
C. Anting-anting
D. Kalung
E. Gelang
G. Gelang kaki

Perabotan rumah tangga

A. kursi tamu/sofa
B. meja
C. kursi
D. tempat tidur
E. bangku

Yang mengadakan

01. Pemerintah Pusat
02. Pemerintah Daerah
03. Kelompok agama
04. Organisasi Politik
05. LSM lokal
06. Donatur swasta
07. Perusahaan/korporasi-korporasi
08. Pemerintah/LSM/donatur asing

EC 21. APAKAH RUMAH TANGGA INI MEMPUNYAI KARTU SEHAT ATAU KARTU SEHAT UNTUK ORANG MISKIN?

01. Ya→
02. Tidak

EC22. SIAPA DALAM RUMAH TANGGA INI YANG MEMPUNYAI KARTU SEHAT ATAU KARTU KESEHATAN UNTUK ORANG MISKIN?(lingkari yang memenuhi)

01. semua anggota rumah tangga
02. Hanya orang dewasa (15 tahun ke atas)
03. Hanya anak-anak (14 tahun ke bawah)
04. ________________________ lain-lain

EC23. APAKAH RUMAH TANGGA INI BERPARTISIPASI DALAM PENGUMPULAN DANA UNTUK PROGRAM KESEHATAN?

01. Ya
02. Tidak
03. Tidak Tahu

EC24. APAKAH RUMAH TANGGA INI PERNAH MENGGUNAKAN SURAT KETERANGAN TIDAK MAMPU?

01. Ya
02.Tidak
03. Tidak tahu

IV. MASYARAKAT DESA/KOMUNITAS

Pertanyaan-pertanyaan berikut ini berkaitan dengan tempat tinggal sekarang dan yang dulu/lama.

C1. NAMA TEMPAT DIMANA BPK/IBU/SDR TINGGAL SEBELUM ERUPSI? ______Nama tempat tinggal lama

C2. APAKAH (NAMA TEMPAT TINGGAL YANG LAMA) SAMA DENGAN TEMPAT KELAHIRANNYA?
02. Ya ________________ 02.Tidak 03. Tidak Tahu

C3. APAKAH TEMPAT TINGGAL LAMA ADALAH SEBUAH:
01. desa __________________ 03. kota besar
02. kota kecil ______________ 04. idak tahu

C4. BERAPA TAHUN BPK/IBU/SDR TINGGAL DITEMPAT LAMA TERSEBUT (NAMA TEMPAT TINGGAL YANG DULU)? _____________ TAHUN

C5.JIKA MENIKAH, BERAPA TAHUN PASANGAN BPK/IBU/SDR TINGGAL DITEMPAT LAMA TERSEBUT (NAMA TEMPAT TINGGAL YANG DULU)? _________________ tahun

C6. KAPAN BPK/IBU/SDR DIPAKSA UNTUK PINDAH KE TEMPAT SEKARANG? (TANGGAL PERTAMA KALI TINGGAL DITEMPAT YANG BARU/SEKARANG)___________ TANGGAL HARI/BULAN

C7. DIMANA BPK/IBU/SDR MENDAPATKAN TEMPAT MENGUNGSI?
01. rumah pribadi keluarga
02. rumah prbadi teman
03. rumah pribadi orang lain
04. tempat peribadatan
05. kantor/sekolah
06. tempat perkemahan
07. Barak pengungsian
08. tempat penampungan sementara/Shelter
09. Tenda
10. ruang terbuka

C8. BERAPA KALI BPK/IBU/SDR DIPINDAHKAN SEJAK PERTAMA KALI DITEMPATKAN (TANGGAL PERTAMA KALI DITEMPATKAN)? ___________________ KALI

c9. DIMANA BPK/IBU/SDR SEKARANG BERTEMPAT TINGGAL? ____________ Nama lokasi penempatan

C10. BERAPA KILOMETER DARI TEMPAT TINGGAL SEKARANG DENGAN DESA ASALNYA DULU?
______________Km

Pengalaman masyarakat desa/komunitas

Pertanyaan-pertanyaan berikut berkaitan dengan pikiran dan perasaan terhadap masyarakat komunitas sekarang dibandingkan dengan masyarakat komunitas yang dulu.

C11. APAKAH BPK/IBU/SDR SEKARANG MERASA SEBAGAI BAGIAN DARI MASYARAKAT SETEMPAT?
01. Ya 02. Tidak

C12. PADA SKALA 1 SAMPAI DENGAN 5, SKALA 1 MENUNJUKKAN "SANGAT TIDAK BAHAGIA" DAN SKALA 5 MENUNJUKKAN "SANGAT BAHAGIA", AMBIL SECARA BERSAMA-SAMA, BAGAIMANA BPK/IBU/SDR AKAN MENGATAKAN HARI-HARI BPK/IBU/SDR? (lingkari nomor yang terbaik menjelaskan apa yang bpk/ibu/sdr rasakan)

Sangat tidak bahagia 1 2 3 4 5 Sangat bahagia

C13. SEBERAPA BAIK BPK/IBU/SDR MERASAKAN BAHWA BPK/IBU/SDR SUDAH COCOK DENGAN MASYARAKAT KOMUNITASNYA YANG SEKARANG?

buruk 1 2 3 4 5 Sangat baik

C14. SEBERAPA BAIK BPK/IBU/SDR MERASA BAHWA BPK/IBU/SDR SUDAH COCOK DENGAN MASYARAKAT KOMUNITAS LAMANYA?

buruk 1 2 3 4 5 Sangat baik

C15. SEBERAPA BANYAK BPK/IBU/SDR MEMILIKI KESAMAAN DENGAN KEBANYAKAN ORANG DALAM MASYARAKAT KOMUNITAS BPK/IBU/SDR?

Tidak ada 1 2 3 4 5 Segalanya

C16. SEBERAPA BANYAK BPK/IBU/SDR MEMPUNYAI KESAMAAN DENGAN KEBANYAKAN ORANG DALAM MASYARAKAT KOMUNITASNYA YANG DULU?

Tidak ada 1 2 3 4 5 Segalanya

C17. SEBERAPA PUAS BPK/IBU/SDR TINGGAL DENGAN MASYARAKAT KOMUNITAS BPK/IBU/SDR SEKARANG?

Tidak puas 1 2 3 4 5 Sangat puas

C18. SEBERAPA PUAS BPK/IBU/SDR TINGGAL DENGAN MASYARAKAT KOMUNITAS BPK/IBU/SDR YANG DULU?

Tidak puas 1 2 3 4 5 Sangat puas

C19. BIARKAN BPK/IBU/SDR MEMBAYANGKAN MASYARAKAT YANG IDEAL YANG BPK/IBU/SDR INGINKAN TINGGAL BERSAMA. DIMANA BPK/IBU/SDR AKAN MENILAI MASYARAKAT YANG SEKARANG TINGGAL BERSAMANYA DIBANDINGKAN DENGAN MASYARAKAT YANG DIIDEALKAN?

Paling buruk 1 2 3 4 5 Paling baik

C20. DIMANA BPK/IBU/SDR MENILAI MASYARAKAT KOMUNITASNYA YANG DULU DIBANDINGKAN DENGAN MASYARAKAT IDEALNYA?

Paling buruk 1 2 3 4 5 Paling baik

C21. BERAPA PERSEN DARI SANAK SAUDARA BPK/IBU/SDR (DAN KELUARGA PASANGAN BPK/IBU/SDR) DAN DENGAN MASYARAKAT KOMUNITAS YANG SEKARANG DALAM JARAK 50 KM?

☐ 0 sampai 25% ☐ 26% sampai 50% ☐ 51% sampai 75% ☐ 76% sampai 100%

C22. BERAPA PERSEN DARI SANAK SAUDARA BPK/IBU/SDR (DAN KELUARGA PASANGAN BPK/IBU/SDR) DAN DENGAN MASYARAKAT KOMUNITAS YANG DULU DALAM JARAK 50 KM?

☐ 0 sampai 25% ☐ 26% sampai 50% ☐ 51% sampai 75% ☐ 76% sampai 100%

C23. KIRA-KIRA BERAPA PERBANDINGAN DARI TEMAN-TEMAN DEKAT YANG TINGGAL DALAM MASYARAKAT KOMUNITAS SEKARANG?

01. Tidak ada atau sangat amat sedikit dari bpk/ibu/sdr
02. Kurang dari setengah dari bpk/ibu/sdr
03. Kira-kira setengahnya
04. Kebanyakan dari bpk/ibu/sdr
05. Semuanya teman dekat

C24. KIRA-KIRA BERAPA PERBANDINGAN ORANG DEWASA YANG TINGGAL DI (NAMA TEMPAT PENEMPATAN YANG SEKARANG) YANG AKAN BPK/IBU/SDR KATAKAN TAHU NAMANYA?

01. Tidak ada atau sangat amat sedikit dari bpk/ibu/sdr
02. Kurang dari setengah dari bpk/ibu/sdr
03. Kira-kira setengahnya
04. Kebanyakan dari bpk/ibu/sdr
05. Semuanya teman dekat

C25. KIRA-KIRA BERAPA PERBANDINGAN DARI HUBUNGAN BPK/IBU/SDR DENGAN ORANG-ORANG YANG BPK/IBU/SDR KETAHUI HANYA DALAM PERTEMUAN RESMI?

01. Tidak ada atau sangat amat sedikit dari bpk/ibu/sdr
02. Kurang dari setengah dari bpk/ibu/sdr
03. Kira-kira setengahnya
04. Kebanyakan dari bpk/ibu/sdr
05. Semuanya teman dekat

C26. MANA DARI SALAH SATU PERNYATAAN DI BAWAH INI YANG TERBAIK MENJELASKAN SEBERAPA BAIK BPK/IBU/SDR SUKA TINGGAL DI MASYARAKAT KOMUNITAS YANG SEKARANG DIBANDINGKAN DENGAN MASYARAKAT YANG DULU (TEMPAT TINGGAL BPK/IBU/SDR YANG LAMA)?
(silahkan tandai satu jawaban saja)

☐ Bpk/ibu/sdr akan melakukan apapun sebisa mungkin untuk tetap tinggal di sini. Bpk/ibu/sdr tidak ingin kembali ketempat yang dulu.
☐ Bpk/ibu/sdr akan menolak kembali ketempatnya jika bpk/ibu/sdr diharuskan untuk itu.
☐ Itu tidak membuatnya berbeda apakah bpk/ibu/sdr tinggal di sini atau di tempat aslinya dulu.
☐ Bpk/ibu/sdr akan lebih suka tinggal dilingkungan masyarakat komunitasnya yang dulu.
☐ Bpk/ibu/sdr akan melakukan apapun untuk meninggalkan masyarakat komunitasnya yang sekarang untuk kembali ke masyarakat komunitasnya yang dulu.

<u>Perasaan tentang masyarakat komunitas</u>

Pertanyaan-pertanyaan berikut ini menanyakan tentang masyarakat komunitas yang sekarang atau yang baru.

Pewawancara: bacalah tiap-tiap pernyataan dengan keras. Kemudian silahkan bpk/ibu/sdr mengatakannya pada saudara apakah bpk/ibu/sdr Sangat setuju dengan pernyataan itu, apakah setuju atau tidak setuju atau bahkan sama sekali tidak setuju dengan pernyataan tersebut.

C27. SAUDARA DAPAT MEMPEROLEH APAPUN YANG SAUDARA INGINKAN DALAM [MASYARAKAT KOMUNITAS INI NAMANYA..].

Sangat setuju 1 2 3 4 5 Tidak setuju

C28. [MASYARAKAT KOMUNITAS SEBUT NAMANYA] MEMBANTU SAUDARA MEMENUHI KEBUTUHAN-KEBUTUHAN SAUDARA.

Sangat setuju 1 2 3 4 5 Tidak setuju

C29. SAUDARA MERASA SEBAGAI ANGGOTA DARI [MASYARAKAT KOMUNITAS SEBUT NAMANYA].

Sangat setuju 1 2 3 4 5 Tidak setuju

C30. SAUDARA MENJADI BAGIAN MILIK DARI [MASYARAKAT KOMUNITAS SEBUTKAN NAMANYA].

Sangat setuju 1 2 3 4 5 Tidak setuju

C31. SAUDARA MEMPUNYAI SESUATU YANG INGIN DIKATAKAN TENTANG APA YANG SEDANG TERJADI DALAM MASYARAKAT KOMUNITAS INI [SEBUTKAN NAMA KOMUNITASNYA].

Sangat setuju 1 2 3 4 5 Tidak setuju

C32. ORANG-ORANG DALAM [KOMUNITAS SEBUTKAN NAMANYA] SANGAT BAIK DALAM MEMPENGARUHI SATU SAMA LAIN.

Sangat setuju 1 2 3 4 5 Tidak setuju

C33. SAUDARA MERASA BERHUBUNGAN DEKAT DENGAN [MASYARAKAT KOMUNITAS INI SEBUTKAN NAMANYA].

Sangat setuju 1 2 3 4 5 Tidak setuju

C34. SAUDARA MEMPUNYAI IKATAN YANG ERAT DENGAN ORANG LAIN DALAM [MASYARAKAT KOMUNITAS SEBUTKAN NAMANYA].

Sangat setuju 1 2 3 4 5 Tidak setuju

Partisipasi masyarakat

Bagian ini menanyakan tentang masyarakat komunitasnya atau kegiatan pemerintah dan program-program yang mungkin telah dilakukan dalam masyarakat yang sekarang berkaitan dengan bencana alam.

Lingkari jawaban yang benar untuk tiap-tiap pilihan

C35. APAKAH KEPEMIMPINAN FORMAL DESA TELAH DIORGANISIR ATAU DITUNJUK DALAM MASYARAKAT KOMUNITAS SEKARANG INI (DITEMPAT TINGGAL SEKARANG INI) SEJAK ERUPSI?

Kepala Desa.. 1. Ya 2. Tidak 3. Tidak tahu
Aparat Desa .... 1. Ya 2. Tidak 3. Tidak tahu
Perwakilan lembaga desa atau
Lembaga Musyawarah Desa (LMD) .... 1. Ya 2. Tidak 3. Tidak tahu
Lembaga Ketahanan Masyarakat Desa (LKMD) .... 1. Ya 2. Tidak 3. Tidak tahu

C36. ADAKAH DARI BEBERAPA UNIT DESA DIBENTUK /DITUNJUK DALAM MASYARAKAT YANG SEKARANG SEJAK ERUPSI?

Desa kecil .... 1. Ya 2. Tidak 3. Tidak tahu
Ketetanggaan.. 1. Ya 2. Tidak 3. Tidak tahu
Rukun warga .... 1. Ya 2. Tidak 3. Tidak tahu
Rukun Tetangga .... 1. Ya 2. Tidak 3. Tidak tahu
Kepala rumah tangga (KK) .... 1. Ya 2. Tidak 3. Tidak tahu

C37. ADAKAH BEBERAPA PERTEMUAN ATAU RAPAT-RAPAT MASYARAKAT KOMUNITAS TELAH DISELENGGARAKAN SEJAK ERUPSI?

Kepala Rumah Tangga (KK) 1. Ya 2. Tidak 3. Tidak tahu
RT.. 1. Ya 2. Tidak 3. Tidak tahu
RW .... 1. Ya 2. Tidak 3. Tidak tahu
Desa .... 1. Ya 2. Tidak 3. Tidak tahu
Kecamatan............................... 1. Ya 2. Tidak 3. Tidak tahu
LMD.. 1. Ya 2. Tidak 3. Tidak tahu
LKMD..................................... 1. Ya 2. Tidak 3. Tidak tahu

C38. APAKAH ADA PEKERJA SUKARELA YANG DIORGANISIR UNTUK MEMBANTU MEMPERBAIKI KONDISI MASYARAKAT KOMUNITAS YANG DULU?

1. Ya 2. Tidak 3. Tidak tahu

C39. JIKA YA, APAKAH BPK/IBU/SDR BERPARTISIPASI DALAM UPAYA-UPAYA INI?

1. Ya 2. Tidak

C40. APAKAH ADA BEBERAPA TENAGA KERJA SUKARELA YANG DIORGANISIR UNTUK MEMBANTU MEMPERBAIKI KONDISI MASYARAKAT KOMUNITAS YANG SEKARANG?

1. Ya 2. Tidak 3. Tidak tahu

C41. JIKA YA, APAKAH BPK/IBU/SDR BERPARTISIPASI DALAM BEBERAPA UPAYA-UPAYA PERBAIKAN INI?

1. Ya 2. Tidak

C42. ADAKAH SUATU KEGIATAN-KEGIATAN KEAGAMAAN (SEPERTI PERKUMPULAN DOA BERSAMA) YANG TELAH DIORGANISIR DALAM MASYARAKAT KOMUNITAS SEKARANG INI?

1. Ya 2. Tidak 3. Tidak tahu

C43. JIKA YA, APAKAH BPK/IBU/SDR BERPARTISIPASI DALAM BEBERAPA KEGIATAN-KEGIATAN INI SEMENJAK ERUPSI?

1. Ya 2. Tidak 3. Tidak tahu

Bagian ini mengenai arisan dimana bpk/ibu/sdr bisa atau tidak bisa berpartisipasi sejak ditempatkan dilokasi yang sekarang ini.

C44. APAKAH BPK/IBU/SDR BERPARTISIPASI DALAM KEGIATAN ARISAN SEJAK ERUPSI?

1. Ya 2. Tidak

C45. JENIS ARISAN APA BPK/IBU/SDR IKUT TERLIBAT SEJAK ERUPSI?

01. Kantor
02. RT
03. RW
04. Desa
05. Ikatan Ibu-Ibu (isteri PNS/tentara)
06. PKK
07. Pasar
08. Keluarga
09. Kelompok agama
10. Teman atau yang lainnya
11. Pensiunan
12. Kelompok tani
13. Kelompok pemuda
14. Arisan sepeda motor

C46. SECARA KESELURUHAN, BERAPA KALI BPK/IBU/SDR MENGIKUTI PERTEMUAN ARISAN SEJAK ERUPSI?___________________kali pertemuan

C47. SECARA KESELURUHAN, UNTUK SEMUA JENIS ARISAN DIMANA BPK/IBU/SDR IKUT SEJAK ERUPSI, BERAPA JUMLAH UANG YANG DIKELUARKAN UNTUK ITU?

1. □□,□□□,□□□ Rp. 2. Tidak tahu

C48. SECARA KESELURUHAN, DARI SEMUA ARISAN DIMANA BPK/IBU/SDR IKUT DALAM 12 BULAN TERAKHIR INI, BERAPA JUMLAH UANG YANG BPK/IBU/SDR TERIMA?

1. □□,□□□,□□□ Rp. 2. Tidak tahu

Silahkan ditandai pernyataan yang tepat atau cocok yang terbaik menjelaskan pendapat bpk/ibu/sdr dengan menggunakan skala 1 sampai dengan 5 dimana skala 1 berarti "sangat tidak setuju" dan skala 5 berarti "sangat setuju."

	**Sangat tidak setuju**<br>**1**	**Agak tidak setuju**<br>**2**	**Biasa saja**<br>**3**	**Agak setuju**<br>**4**	**Sangat setuju**<br>**5**
Masa depan masyarakat komunitas ini nampaknya sangat cerah.	☐	☐	☐	☐	☐
Warga masyarakat komunitas ini berpartisipasi dalam semua urusan masyarakat.	☐	☐	☐	☐	☐
Warga masyarakat komunitas ini sangat menerima warga baru yang menduduki posisi pimpinan.	☐	☐	☐	☐	☐
Organisasi-organisasi dan kelompok-kelompok tertarik pada apa yang terbaik untuk semuanya.	☐	☐	☐	☐	☐
Orang-orang dalam komunitas ini bekerja bersama-sama untuk menyelesaikan semua pekerjaan yang harus dikerjakan.	☐	☐	☐	☐	☐
Bpk/ibu/sdr sangat aktif dalam kegiatan-kegiatan perbaikan masyarakat setempat.	☐	☐	☐	☐	☐
Konflik tidak pernah ada antara orang-orang dalam kelompok masyarakat ini.	☐	☐	☐	☐	☐
Bpk/ibu/sdr menaruh kepercayaan pada orang-orang di masyarakat ini.	☐	☐	☐	☐	☐
Orang-orang dalam masyarakat ini mempunyai kesamaan nilai-nilai budaya dan tradisi.	☐	☐	☐	☐	☐

V. KESEHATAN FISIK DAN MENTAL

Bagian ini mengenai kesehatan fisik dan mental dari yang diwawancarai.

H1. MINTALAH KEPADA BPK/IBU/SDR UNTUK MENILAI KESEHATAN FISIK BPK/IBU/SDR DARI SKALA 1 SAMPAI DENGAN SKALA 5 DIMANA SKALA 1 MENUNJUKKAN "SANGAT BAIK" DAN SKALA 5 MENUNJUKKAN "SANGAT BURUK."

Sangat baik 1 2 3 4 5 Sangat buruk

H2. MINTALAH KEPADA BPK/IBU/SDR UNTUK MENILAI KESEHATAN MENTAL BPK/IBU/SDR DARI SKALA 1 SAMPAI DENGAN SKALA 5 DIMANA SKALA 1 MENUNJUKKAN "SANGAT BAIK" DAN SKALA 5 MENUNJUKKAN "SANGAT BURUK."

Sangat baik 1 2 3 4 5 Sangat buruk

H3. TEMUKAN JIKA BPK/IBU/SDR SEDANG MENGALAMI MASALAH KESEHATAN FISIK ATAU MENTAL?

01. YA 02. TIDAK

H4.

Jika bpk/ibu/sdr sedang mengalami kesehatan fisik atau mental, mintalah bpk/ibu/sdr untuk menjelaskan secara khusus dan menuliskannya dilembar kosong yang tersedia

A. MASALAH-MASALAH KESEHATAN MENTAL

1. ____
2. ____
3. ____

B. MASALAH-MASALAH KESEHATAN FISIK

1. ____
2. ____
3. ____

H5. SELAMA BULAN YANG LALU, TEMUKAN BERAPA KALI BPK/IBU/SDR MELAKUKAN KONSULTASI DENGAN KONSELING ATAU AHLI KESEHATAN ATAU PSIKOLOG UNTUK MENDAPATKAN NASEHAT ATAU PERAWATAN?

_______________ hari

H6. SEBERAPA DEKATKAH TEMPAT KONSULTASI KESEHATAN ATAU TEMPAT UNTUK PENYEMBUHAN TERSEBUT?_________________ Km

H7.

Pertanyaan-pertanyaan berikut ini menanyakan tentang perasaan dan pikiran bpk/ibu/sdr selama bulan yang lalu. Dalam setiap kasus, silahkan ditunjukkan dengan melingkari angka, berapa sering dia merasa atau berpikir dalam hal tertentu.

0 = tidak pernah 1 = hampir tidak pernah 2 = kadang-kadang 3 = cukup sering 4 = sangat sering sekali

a. Pada bulan lalu, seberapa sering saudara merasa sedih karena sesuatu yang tidak diharapkan terjadi? **0 1 2 3 4**

b. Pada bulan lalu, seberapa sering saudara merasa bahwa saudara tidak dapat menguasai hal-hal penting dalam hidup saudara? **0 1 2 3 4**

c. Pada bulan lalu, seberapa sering saudara merasa gelisah dan tertekan? **0 1 2 3 4**

d. Pada bulan lalu, seberapa sering saudara merasa yakin tentang kemampuan saudara menangani masalah-masalah pribadi saudara? **0 1 2 3 4**

e. Pada bulan lalu, seberapa sering saudara merasa segala sesuatu berjalan sesuai dengan cara **0 1 2 3 4**

saudara?

f. Pada bulan lalu, seberapa sering saudara mendapatkan bahwa saudara tidak dapat menangani segala sesuatu seperti yang harus saudara lakukan? **0 1 2 3 4**

0 1 2 3 4

g. Pada bulan lalu, seberapa sering saudara dapat mengatasi gangguan-gangguan dalam kehidupan saudara**?** **0 1 2 3 4**

h. Pada bulan lalu, seberapa sering saudara merasa bahwa saudara ada di atas segala-galanya?

0 1 2 3 4

i. Pada bulan lalu, seberapa sering saudara dibuat marah karena sesuatu di luar kendali saudara?

j. Pada bulan lalu, seberapa sering saudara merasa kesulitan-kesulitan menumpuk begitu banyak yang mana saudara sendiri tidak bisa mengatasinya? **0 1 2 3 4**

H8.

Berikut ini, biarkan bpk/ibu/sdr berpikir tentang seminggu yang lalu. Mintalah pada bpk/ibu/sdr untuk mengatakan pada anda berapa hari dalam seminggu bpk/ibu/sdr: (lingkari satu untuk masing-masing pernyataan)

	**Tidak ada**	**1 hari**	**2 hari**	**3 hari**	**4 hari**	**5 hari**	**6 hari**	**7 hari**
Merasa terganggu oleh sesuatu yang biasanya tidak mengganggunya?	0	1	2	3	4	5	6	7
Merasa tidak suka makan, selera makannya hilang?	0	1	2	3	4	5	6	7
Merasa bpk/ibu/sdr tidak dapat menghilangkan rasa sedih bahkan dengan bantuan keluarga atau teman sekalipun?	0	1	2	3	4	5	6	7
Mempunyai masalah untuk tetap berpikir pada apa yang bpk/ibu/sdr kerjakan?	0	1	2	3	4	5	6	7
Merasa depresi?	0	1	2	3	4	5	6	7
Merasa segala sesuatu yang dikerjakan adalah hasil dari suatu usaha?	0	1	2	3	4	5	6	7
Merasa ketakutan?	0	1	2	3	4	5	6	7
Tidur tidak nyenyak?	0	1	2	3	4	5	6	7
Bicara kurang dari biasanya?	0	1	2	3	4	5	6	7
Merasa kesepian?	0	1	2	3	4	5	6	7

Merasa sedih?	0	1	2	3	4	5	6	7
Merasa anda tidak dapat berjalan maju?	0	1	2	3	4	5	6	7
Berpikir tidak ada lagi yang berharga?	0	1	2	3	4	5	6	7
Khawatir tentang sesuatu atau seseorang?	0	1	2	3	4	5	6	7

H9.

Berikut ini, silahkan bpk/ibu/sdr menjelaskan pendapat bpk/ibu/sdr untuk tiap-tiap pernyataan yang berkisar antara 1 hingga 5, dimana 1 menunjukkan "sangat tidak setuju" sampai dengan 5 menunjukkan "sangat setuju."

	**Sangat tidak setuju**<br>**1**	**agak kurang setuju**<br>**2**	**Biasa saja**<br>**3**	**Agak setuju**<br>**4**	**Sangat setuju sekali**<br>**5**
Saya merasa apa yang terjadi dalam hidup saya sepenuhnya ditentukan oleh kekuatan pihak lain.	☐	☐	☐	☐	☐
Ketika saya membuat rencana, saya hampir yakin akan berjalan.	☐	☐	☐	☐	☐
Ketika saya mendapatkan apa yang saya inginkan, itu karena keberuntungan.	☐	☐	☐	☐	☐
Meskipun saya mungkin memiliki kemampuan yang baik, saya tidak akan diberikan tanggungjawab kepemimpinan tanpa meminta kekuasaan untuk jabatan tersebut.	☐	☐	☐	☐	☐
Seberapa banyak teman-teman yang saya punyai tergantung pada seberapa baik saya sebagai pribadi.	☐	☐	☐	☐	☐
Saya sering menemukan bahwa apa yang akan terjadi pasti akan terjadi.	☐	☐	☐	☐	☐
Kehidupan utama saya dikuasai oleh kekuatan orang lain.	☐	☐	☐	☐	☐
Tidak selalu bijak untuk saya merencanakan terlalu jauh ke depan karena banyak hal berubah dari sesuatu yang baik menjadi ketidak	☐	☐	☐	☐	☐

beruntungan.					
Ketika saya mendapatkan apa yang saya inginkan, biasanya karena saya telah bekerja keras untuk itu.	☐	☐	☐	☐	☐
Mendapatkan sesuatu yang saya inginkan butuh menyenangkan siapa saja yang ada di atas saya.	☐	☐	☐	☐	☐
Apakah saya jadi atau tidak seorang pemimpin tergantung pada jika saya cukup beruntung berada di tempat dan waktu yang tepat.	☐	☐	☐	☐	☐
Saya cukup menentukan apa yang akan terjadi dalam kehidupan saya.	☐	☐	☐	☐	☐

H10.

Silahkan tunjukkan pada tiap-tiap pernyataan berikut, yang sesuai dengan kondisi bpk/ibu/sdr seperti yang bpk/ibu/sdr rasakan saat ini. Silahkan dilingkari sesuai dengan jawaban yang paling bpk/ibu/sdr anggap tepat.

	**Ya!**	**Ya**	**Kurang lebih**	**tidak**	**tidak!**
a. selalu ada seseorang dimana saya dapat berbicara tentang masalah sehari-hari.	☐	☐	☐	☐	☐
b. Saya kehilangan teman yang sungguh-sungguh dekat.	☐	☐	☐	☐	☐
c. saya mengalami secara keseluruhan kehampaan.	☐	☐	☐	☐	☐
d. ada banyak orang dimana saya dapat bergantung ketika saya mempunyai masalah.	☐	☐	☐	☐	☐
e. saya kehilangan kesenangan berkumpul dengan orang lain.	☐	☐	☐	☐	☐
f. saya menemukan keterbatasan teman-teman dan kenalan disekitar saya.	☐	☐	☐	☐	☐
g. ada banyak orang yang dapat saya percayai secara keseluruhan.	☐	☐	☐	☐	☐
h. saya merasa kehilangan orang-orang disekitar saya.	☐	☐	☐	☐	☐
i. saya sering merasa ditolak.	☐	☐	☐	☐	☐
j. Saya dapat memanggil teman-teman saya kapanpun saya membutuhkan bpk/ibu/sdr.	☐	☐	☐	☐	☐

Ini merupakan serangkaian pertanyaan terakhir berkaitan dengan pendapat responden erhadap seluruh kualitas hidup bpk/ibu/sdr.

H11. SILAHKAN BPK/IBU/SDR MEMBAYANGKAN ENAM TINGKATAN DARI YANG PALING RENDAH (TINGKATAN PERTAMA), MENUNJUKKAN KEADAAN PALING MISKIN DARI SESEORANG, DAN PADA TINGKATAN PALING TINGGI (TINGKATAN KEENAM) YANG MENUNJUKKAN KEADAAN ORANG YANG PALING KAYA, PADA TINGKATAN MANAKAH KONDISI BPK/IBU/SDR SENDIRI SAAT INI?

TERMISKIN 1	2	3	4	5	TERKAYA 6

H12. PADA TINGKATAN MANAKAH KONDISI BPK/IBU/SDR SEBELUM BENCANA ERUPSI?

TERMISKIN 1	2	3	4	5	TERKAYA 6

H13. PADA TINGKAT MANAKAH BPK/IBU/SDR MENGHARAPKAN KEADAAN BPK/IBU/SDR DALAM WAKTU LIMA TAHUN MENDATANG?

TERMISKIN 1	2	3	4	5	TERKAYA 6

VI. TABEL ANGGOTA RUMAH TANGGA

Anggota rumah tangga	Umur	Status Perkawinan	Agama	Suku Bangsa	Pendidikan
1.		□□	□□	□□	□□
2.		□□	□□	□□	□□
3.		□□	□□	□□	□□
4.		□□	□□	□□	□□
5.		□□	□□	□□	□□
6.		□□	□□	□□	□□
7.		□□	□□	□□	□□
8.		□□	□□	□□	□□
9.		□□	□□	□□	□□

Kode-kode

Marital Status

01. Tidak menikah
02. Menikah
03. Berpisah
04. Bercerai hidup
05. Bercerai mati

Agama

01. Islam
02. Protestan
03. Katolik
04. Hindu
05. Buddha
07. Konfucu
08. lain-lain

Suku Bangsa

01. Java

02. Sunda
03. Bali
04. Batak
05. Bugis
06. Cina
07. Madura
08. Sasak
09. Minang
10. Banjar
11. Bima-Dompu
12. Makasar
13. Nias
14. Palembang
15. Sumbawa
16. Toraja
17. Betawi
18. Dayak
19. Melayu
20. Komering
21. Ambon
22. Manado
23. Aceh
25. Sumatera selatan
26. Banten
27. Cirebon
28. Gorontalo
29. Kutai
30. Lain-lain

Pendidikan
01. SD
02. SMP
03. SMA
04. Akademi/D1/D2/D3/
Sarjana Muda
05. Sarjana (S1)
06. S2
07. S3
08. Tidak tahu
09. Lain-lain
