# Supplementary material for: Community recovery after a natural disaster: Core data from a survey of communities affected by the 2010 Mt. Merapi eruptions in Central Java, Indonesia
Source: Data Brief. 2020 Jul 19;32:106040. doi: 10.1016/j.dib.2020.106040 (PMC7393468; doi:10.1016/j.dib.2020.106040)
Supplement: Supplementary file 3 [file mmc3.pdf]

CODE BOOK DATA OF MERAPI

I. GENERAL INFORMATION

Note: The data in this section were gathered using survey questions primarily drawn from or adapted to maintain consistency with questions in the Indonesia Demographic and Health Survey (IDHS) and the Indonesian Family Life Survey (IFLS) [1, 2].

No.	Variable	Label	Value		
1	No	Respondent's Number	1 - 400		
2	Nares2	Name of Respondent	**-Not Publicly Available-**		
3.	Reg3	Respondent's Region/Municipality	1. Sleman		
4.	Prov4	Respondent's Province	1. DIY		
5.	City5	Respondent's City	1. Sleman		
6.	Nacor6	Name of coordinator	1. Ir. Christine Sri Widi P. MP<br>2. Drs. Hardjono, M.Si<br>3. Ir. Rini Dorojati, MS<br>4. Drs. RY Gatot, M.Si<br>5. Yuli Setyowati, S.IP., M.Si	6. Dra. Nuraini Dwi Astuti, MP<br>7. Dra. Suharti, M.Si<br>8. Dra. Widati, lil rer reg<br>9. Rr. Leslie Retno A, Ph.D<br>10. Drs. Supardal, M.Si	
7.	Dist7	Respondent's District/Subdistrict	1. Turi<br>2. Ngemplak<br>3. Pakem<br>4. Cangkringan		
8.	Vill8	Respondent's Village	1. Wonokerto<br>2. Girikerto<br>3. Sindumartani<br>4. Umbulharjo<br>5. Kepuharjo	6. Argomulyo<br>7. Glagaharjo<br>8. Wukirsari<br>9. Purwobinangun<br>10. Hargobinangun	
9.	Hamlet9	Respondent's Hamlet or Dusun	1. Tunggularum<br>2. Sari (Randualas)<br>3. Garongan<br>4. Kembang<br>5. Ngandong<br>6. Nganggring<br>7. Surodadi<br>8. Karang Gawang<br>9. Plumbon<br>10. Jelapan<br>11. Kentingan<br>12. Pencar	21. Shelter Gondang II<br>22. Shelter Gondang III<br>23. Bronggang<br>24. Gadingan<br>25. Losari<br>26. Kuwang<br>27. Shelter Srunen<br>28. Shelter Banjarsari<br>29. Shelter Ngancar<br>30. Besalen<br>31. Shelter Gondangsari<br>32. Bulaksalak	

			13. Tambaan<br>14. Kayen<br>15. Shelter Kinahrejo<br>16. Pangukrejo<br>17. Gondang<br>18. Gambretan<br>19. Shelter mandiri<br>20. Shelter Gondang I	33. Turgo<br>34. Ngepring<br>35. Ngelosari<br>36. Kemiri<br>37. Kaliurang Barat<br>38. Kaliurang<br>39. Kaliurang Timur<br>40. Boyong
10.	Rcom10	Respondent's Community Solidarity (RW)	1. RW 1<br>2. RW 2<br>3. RW 3<br>4. RW 4<br>5. RW 5<br>6. RW 6<br>7. RW 7<br>8. RW 8<br>9. RW 9<br>10.RW 10	11. RW 11<br>12. RW 12<br>13. RW 13<br>14. RW 14<br>15. RW 15<br>16. RW 16<br>17. RW 17<br>18. RW 18<br>19. RW 19
11.	Rnieg11	Respondent's Neighbor Solidarity (RT)	1. RT 1<br>2. RT 2<br>3. RT 3<br>4. RT 4<br>5. RT 5	6. RT 6<br>7. RT 7<br>8. RT 8<br>9. RT 9
12.	Shen12	Shelter name	0. Non shelter<br>1. Kentingan<br>2. Kinahrejo<br>3. Pangukrejo/Plosokerep<br>4. Gondang<br>5. Shelter Mandiri<br>6. Gondang 1	7. Gondang 2<br>8. Gondang 3<br>9. Srunen<br>10. Banjarsari<br>11. Ngancar<br>12. Gondangsari
13.	Scso13	Shelter Community Solidarity (RW)	0. None<br>1. RW 1<br>2. RW 2<br>3. RW 3<br>4. RW 4<br>5. RW 5<br>6. RW 6<br>7. RW 7<br>8. RW 8	

14.	Snei14	Shelter Neighbor Solidarity (RT)	0. None<br>1. RT 1<br>2. RT 2<br>3. RT 3<br>4. RT 4<br>5. RT 5	
15.	Nain15	Name of the interviewer	1.<br>2.<br>3.<br>4.<br>5.<br>6.<br>7.<br>8.<br>9.<br>10.<br>11.<br>12.<br>13.<br>14.<br>15.<br>16.<br>17.<br>18.<br>19.<br>20.	21.<br>22.<br>23.<br>24.<br>25.<br>26.<br>27.<br>28.<br>29.<br>30.<br>31.<br>32.<br>33.<br>34.<br>35.<br>36.<br>37.<br>38.<br>39.<br>40.
16.	Datin16	Date Interview		
17.	Timst17	Time Start Interview		
18.	Timfh18	Time Finish Interview		
19.	Resta19	Respondent's Status in HH		

20. Age20 Respondent's Age {1, 17}...

21. Resta21 Respondent's Marital Status {1, Not married}...

22. Sex22 Respondent's Sex {1, Male}...

23. Ethn23 Respondent's Ethnicity {1, Javanese}...

24. Reli24 Respondent's Religion {1, Islam}...

25. Edu25 Respondent's Education {0, Not yet go to school}...

II. Event

Note: The data in this section were gathered using survey questions primarily drawn from or adapted to maintain consistency with questions that are consistent with those found in social science research aimed at measuring the social correlates of a disaster [3, 4, 5].

26. Disf26 Flood {1, Flood}...

27. Disl27 Landslide {1, Landslide}...

28. Dism28 Mudflow {1, Mudflows}...

29. Diser29 Eruption {1, Eruption}...

30. Disv30 Volcanic {1, Volcanic}...

31. Disq31 Earthquake {1, Earthquake}...

32. Dist32 Tsunami {1, Tsunami}...

33. Disw33 Windstorm {1, Windstorm}...

34. Disff34 Forest Fire {1, Forest Fire}...

35. Disfir35 Fire {1, Fire}...

36. Tyhc36 Type disaster Head Cloud {1, Head cloud}...

37. Tyer37 Type disaster eruption {1, Eruption}...

38. Tyds38 Type disaster dust {1, Dust}...

39. Tylf39 Type disaster lava flow {1, Lava flow}...

40. Tycf40 Type disaster cold flow {1, Cold flow}...

41. Tywc41 Type disaster water contamination {1, Water contamination}...

42. Tym42 Type disaster mud flow {1, Mudflow}...

43. Tyls43 Type disaster land slide {1, Landslide}...

44. Tywf44 Type disaster forest fire {1, Forest fire}...

45. Tyep45 Type disaster electricity power outage {1, Electricity power outage}...

46. Tyfd46 Type disaster floods {1, Floods}...

47. Mefv47 Member of family being victim {1, No}...

48. Nufv48 Number of family member being victim {0, None}...

49. Relfv49 Relation to the victim {1, Child, aunty/uncle's children}...

50. Mefin50 Member of family seriously injured {1, No}...

51. Nuin51 Number of family member seriously injured {0, None}...

52. Remin52 Relation to the seriously injured {1, Child}...

53. Peimp53 Perception to the disaster impacts {1, Minimal damage}...

54. Toblo54 Total Rupiah business assets lost {0, None}...

55. Perlos55 Percentages total business assets lost {0, None}...

56. Tonblo56 Total Rupiah non business assets lost {0, None}...

57. Penblo57 Percentages total non business assets lost {0, None}...

58. Redes58 Old resident house ruined or destroyed {1, Not damaged}...

59. Renov59 Old resident house has renovated or rebuilt {1, Yes}...

60. Albac60 Allow to move back to previous community {1, Yes}...

61. Reprec61 Return to their previous community {1, Yes}...

62. Livtem62 Currently live in temporary housing {1, Yes}...

63. Tytem63 If still living in temporary housing type of place {1, Privately-owned home family}...

64. Dursty64 Duration time stay in shelter or temporary house {0, None}...

65. Fnores65 Family member without resident or shelter to stay {1, Yes}...

66. Typlas66 Type place to stay {1, Privately-owned home family}...

67. Dufsta67	Duration time family member to stay in temporary house	{0, 0 None}...
68. Wrat68	Fear wrath of natural disaster	{1, strongly agree}...
69. NdisA69	Natural disaster are a form of Allah punishment	{1, Strongly agree}...
70. Natlcy70	Natural disaster is a part of life cycle	{1, strongly agree}...
71. Leharn71	Learn living harmony with nature	{1, Strongly agree}...
72. Decom72	Feel deep connection with Merapi	{1, Strongly agree}...
73. Copred73	Old community predict natural disaster best	{1, Strongly agree}...
74. Lislop74	Live on the slope better than other safe place	{1, Strongly agree}...
75. Reold75	Return to old community no matter what happen	{1, Strongly agree}...
76. Goprep76	Government conduct preparation prior disaster	{1, Yes}...
77. Goprev77	Government do prevention for disaster	{1, Yes}...
78. Gomit78	Government do mitigation disaster	{1, Yes}...
79. Gores79	Government do response to disaster	{1, Yes}...
80. Gorec80	Government do recovery after disaster	{1, Yes}...
81. Ngprep81	Non-Government conduct preparation prior disaster	{1, Yes}...
82. Ngprev82	Non-Government do prevention for disaster	{1, Yes}...
83. Ngmit83	Non-Government do mitigation disaster	{1, Yes}...
84. Ngres84	Non-Government do response to disaster	{1, Yes}...
85. Ngrec85	Non-Government do recovery after disaster	{1, Yes}...
86. Paprep86	Respondent participate in preparation	{1, Yes}...
87. Paprev87	Respondent participate on prevention	{1, Yes}...
88. Parmit88	Respondent participate on mitigation disaster	{1, Yes}...
89. Pares89	Respondent participate on response to disaster	{1, Yes}...

90. Parec90 Respondent participate on recovery after disaster {1, Yes}...

91. Efprep91 Effective rate of preparation prior disaster {1, Extremely effective}...

92. Efprev92 Effective rate of prevention {1, Extremely effective}...

93. Efmit93 Effective rate of mitigation disaster {1, Extremely effective}...

94. Efres94 Effective rate of response {1, Extremely effective}...

95. Efrec95 Effective rate of recovery {1, Extremely effective}...

96. Prepm96 Preparation methods align with respondent culture {1, Yes}...

97. Prevm97 Prevention methods align with respondent culture {1, Yes}...

98. Mitm98 Mitigation disaster methods align with respondent culture {1, Yes}...

99. Resm99 Response methods align with respondent culture {1, Yes}...

100. Recm100 Recovery methods align with respondent culture {1, Yes}...

101. Qdev101 Quality of development implemented by the Government {1, Excellent}...

102. Qplan102 Quality of planning implemented by the Government {1, Excellent}...

103. Qbul103 Quality of building implemented by the Government {1, Excellent}...

104. Qplac104 Quality of placement implemented by the Government {1, Excellent}...

105. Qcom105 Quality of com-dev continuation implemented by the Government {1, Excellent}...

III. Standard of Living

Note: Variables 106 – 128 were gathered from questions that are consistent with those found in social science research aimed at measuring the social correlates of a disaster [3, 4, 5]. The balance of the data in this section were gathered using survey questions primarily drawn from or adapted to maintain consistency with questions in the IDHS and the IFLS [1, 2].

106. Refin106 Currently receive financial assistance {1, Yes, currently}...

107. Sorfin107 Source of financial assistance {1, Central government}...

108. Tofin108 Total amount of financial assistance {0, None}...

109. Pefin109 Percentage of total finance assistance {0, None}...

110. Aqfin110 Adequate financial assistance scale {1, Very adequate}...

111. Rehel111 Currently receive health care {1, Yes, currently}...

112. Cohel112 Cover appropriate care for their health {1, Complete coverage}...

113. Fahel113 Nearest healthcare facility offer the appropriate care {1, 0}...

114. Quhel114 Overall quality health care rate {1, Excellent}...

115. Refod115 Currently receiving food assistance {1, Yes, currently}...

116. Sofod116 Source of food assistance {0, None}...

117. Adfod117 Adequate food assistance {1, Very adequate}...

118. Ecar118 Essential food carbohydrates not included in food assistance {1, Yes}...

119. Epro119 Essential protein not included in food assistance {1, Yes}...

120. Efru120 Essential fruits not included in food assistance {1, Yes}...

121. Eveg121 Essential vegetables not included in food assistance {1, Yes}...

122. Edai122 Essential dairy not included in food assistance {1, Yes}...

123. Ecoc123 Essential coconut milk not included in food assistance {1, Yes}...

124. Etea124 Essential tea not included in food assistance {1, Yes}...

125. Espi125 Essential spices not included in food assistance {1, Yes}...

126. Relod126 Currently receiving lodging money from Government or Non-Government {1, Yes, currently receiving}...

127. Sorlod127 Source of lodging money {0, None}...

128. Felad128 Feel the assistance given adequate their needs {1, Very adequate}...

129. Owhos129 Ownership status of housing {1, Owned}...

130. Tydwe130 Type of resident dwelling {1, Single unit level}...

131. Aniws131 Human or animal waste surrounding house {1, Yes}...

132. Tras132 Pile of trash surrounding house {1, Yes}...

133. Staw133 Stagnant water surrounding house {1, Yes}...

134. Stabh134 Stable near or under house {1, Yes}...

135. Suven135 Sufficient ventilation {1, Yes}...

136. Yarcle136 Yard well maintenance or cleaned-up {1, Yes}...

137. Kitot137 House has a kitchen outside {1, Yes}...

138. Coslep138 Cooking and sleeping in the same room {1, Yes}...

139. Hsiz139 House size in square meters {1, 24}...

140. Torom140 Total rooms in the house {1, 2}...

141. Sorwat141 Source of main water {1, Pipe water}...

142. Safwat142 Doing something for safe water to drink {1, Boil}...

143. Dywat143 Total days to get access to water{1, Daily}...

144. Locwat144 Location source of drinking water {1, Inside the house}...

145. Timwat145 Longer time to get to the source of drinking water {0, 0 minute}...

146. Distwat146 Distance from home to the source of main water {0, 0 m}...

147. Msowat147 Main source of water for other HH purpose get from the same main water {1, Yes}...

148. Swatop148 Source of water for other HH purpose {1, Pipe water}...

149. Dywtop149 Days to get access to the water for other HH purpose {1, Daily}...

150. Lowop150 Location of water for other HH purpose {1, Inside the house}...

151. Otwop151 Outside the house time to get to the source of water for other HH purpose{0, 0 minute}...

152. Odiwop152 Outside the house the distance from home to the source of water for other HH purpose {0, 0 m}...

153. Hhtol153 Household toilet {1, Private waste septic tank}...

154. Hhsew154 Household drain its sewage {1, Drain age ditch (flowing)}...

155. Rewat155 Receive water assistance {1, Yes, currently}...

156. Purwat156 Purpose of water assistance {0, None}...

157. Swats157 Source of water assistance {1, Central government}...

158. Qalas158 Quality all assistance scale {1, Excellent}...

159. Disgar159 Ways household dispose the garbage {1, Dispose in trash can, collected by sanitation service}...

160. Fodref160 Keep foods in the refrigerator {1, Yes}...

161. Sofire161 Source of fire for cooking {1, Electricity}...

162. Livcon162 Overall living condition scale {1, Excellent}...

163. Elecut163 Electricity utilization {1, Yes}...

164. Sorelc164 Source of electricity {1, Solar}...

165. TV165 Own TV {1, Yes}...

166. Comp166 Own computer {1, Yes}...

167. Intnt167 Internet user before {1, Yes}...

168. Pint168 Place to use internet {1, At home}...

169. Kint169 Kinds of internet using {1, Speak with friends and relatives}...

170. Hphon170 Own home phone {1, Yes}...

171. Tphon171 Times using phone {1, Never}...

172. HP172 Own HP {1, No}...

173. Kushp173 Kinds of HP using {0, None}...

174. HPus174 Using HP for work or communicate with friend or family {1, Work}...

175. Tbyca175 Times to buy calling card {1, Never}...

176. Pbyca176 Place to buy calling card {1, A store in my village}...

177. Trevcal177 Total receiving calls from HP {0, 0}...

178. Disvil178 Distance from home to near village or city {1, 1000}...

179.Tcomm179 Total commuters to near village or city a month {1, 0}...

180. Dismar180 Distance from home to near market {1, 100}...

181. Tmar181 Total markets within 5 km from home {0, None}...

182. Ptrans182 Primary mode of transportation {1, Walking}...

IV. Economics

Note: The data in this section were gathered using survey questions primarily drawn from or adapted to maintain consistency with questions in the IDHS and the IFLS [1, 2].

183. Cuwork183 Currently working {1, Working}...

184. Duwork184 Duration times not working {1, 0}...

185. Hawork185 Have other kind of work at least an hour a week {1, Yes}...

186. Typoc186 Types of occupation {1, Professional technical}...

187. Workfa187 Work on farm or nonfarm {1, Farming}...

188. Swork188 Spouse work {1, Yes}...

189. Snwork189 Spouse not working, she has been employed the last 12 months {1, Yes}...

190. Stywork190 Spouse types of work {0, Not working}...

191. Diswork191 Distance travel to work {0, Not working, stay at home}...

192. Satwork192 Satisfaction with current work {1, Very satisfied}...

193. Incom193 Household monthly income {1, Rp150,000}...

194. Insta194 Income stability {1, Yes}...

195. Fstain195 Factors contribute to reliability stable income {1, Arisan}...

196. Pstain196 Person has the most stable income {1, Male Head of HH}...

197. Pconin197 Person who contribute the most money {1, Male Head of HH}...

198. Rmony198 Receive money from people not living at current residence {1, Yes}...

199. Remony199 Relation to the person who give money {0, None, Nobody}...

200. Tmon200 Total amount of money received from other person {0, None}...

201. Tincomx201 Total income from other external sources {0, None}...

202. Ternfod202 Total earning food {0, None}...

203. Ternfir203 Total earning food in rupiah {0, None}...

204. Tpernf204 Total percentage earning food of income {0, None}...

205. Sernf205 Source of earning food {1, Regional government}...

206. Thsup206 Total household supplies earning {0, None}...

207. Thsupr207 Total household supplies in rupiah {0, None}...

208. Tphsup208 Total percentage household supplies of income {0, None}...

209. Shsup209 Source of household supplies {0, None}...

210. Tfsup210 Total farming supply {0, None}...

211. Tfsupr211 Total farming supplies in rupiah {0, None}...

212. Tpfsup212 Total percentage farming supplies of income {0, None}...

213. Sfsup213 Source of farming supplies {0, None}...

214. Tolis214 Total livestock {0, None}...

215. Tolis215 Total livestock in rupiah{0, None}...

216. Toplis216 Total percentage of livestock of income {0, None}...

217. Stoplis217 Source of livestock {1, Central government}...

218. Fborm218 Family member borrow money from other source past 12 months{1, Yes}...

219. Purbm219 Purpose to borrow money {0, None}...

220. Olivs220 Own livestock {1, Yes}...

221. Ocat221 Own cattle/milk cows/bulls {1, Yes}...

222. Ogoat222 Own goat/sheep {1, Yes}...

223. Ohors223 Own horse/donkeys or mules {1, Yes}...

224. Opig224 Own pig {1, Yes}...

225. Opol225 Own poultry {1, Yes}...

226. Ofish226 Own fishpond {1, Yes}...

227. Oothr227 Own other {1, Yes}...

228. Tolivs228 Total livestock {0, None}...

229. Hstaliv229 Have status ownership livestock {1, Yes}...

230. Livpro230 What livestock provided {0, None}...

231. Livdis231 Provided livestock due to disaster {1, Yes}...

232. Oveh232 Own vehicles {1, Yes}...

233. Ocar233 Own cars/truck {1, Yes}...

234. Omotb234 Own motorboat {1, Yes}...

235. Orowb235 Own rowboats {1, Yes}...

236. Obicy236 Own bicycle {1, Yes}...

237. Omotb237 Own motorbike {1, Yes}...

238. Otract238 Own tractor {1, Yes}...

239. Owatv239 Own water vehicle {1, Yes}...

240. Oandc240 Own animal drawn cart {1, Yes}...

241. Totveh241 Total vehicles {0, None}...

242. Hsoveh242 Have status ownership vehicles {1, Yes}...

243. Wvehp243 What vehicles provided {0, None}...

244. Pvehdi244 Provided vehicles due to disaster {1, Yes}...

245. Ohap245 Own house appliances {1, Yes}...

246. Orad246 Own radio {1, Yes}...

247. Otape247 Own tape recorder {1, Yes}...

248. Otelv248 Own TV {1, Yes}...

249. Orefg249 Own refrigerator {1, Yes}...

250. Osew250 Own sewing or washing machine {1, Yes}...

251. Ovcd251 Own VCD player {1, Yes}...

252. Owhp252 Own HP {1, Yes}...

253. Obatc253 Own battery charger {1, Yes}...

254. Otelp254 Own telephone {1, Yes}...

255. Omobp255 Own mobile phone {1, Yes}...

256. Thap256 Total house appliances {0, None}...

257. Howhap257 Have ownership over house appliances {1, Yes}...

258. Whapro258 What house appliances provided {0, None}...

259. Phapd259 Provided house appliances due to disaster {1, Yes}...

260. Ostol260 Own small tools {1, Yes}...

261. Osaw261 Own saws {1, Yes}...

262. Oaxe262 Own axes {1, Yes}...

263. Omach263 Own machete {1, Yes}...

264. Ofork264 Own fork {1, Yes}...

265. Oplow265 Own plow {1, Yes}...

266. Ohoe266 Own hoe {1, Yes}...

267. Orak267 Own rake {1, Yes}...

268. Tstol268 Total small tools {0, None}...

269. Hons269 Have ownership over small tools {1, Yes}...

270. Stolav270 Small tools provided {1, Central government}...

271. Pstold271 Provided small tools due to disaster {1, Yes}...

272. Ojew272 Own jewelry {1, Yes}...

273. Oring273 Own ring {1, Yes}...

274. Owat274 Own watch {1, Yes}...

275. Oear275 Own earrings {1, Yes}...

276. Oneck276 Own necklace {1, Yes}...

277. Obrac277 Own bracelets {1, Yes}...

278. Oankl278 Own anklets {1, Yes}...

279. Tjew279 Total jewelry {0, None}...

280. Hojew280 Have ownership over jewelry {1, Yes}...

281. Wjepro281 What jewelry provided {0, None}...

282. Pjed282 Provided jewelry due to disaster {1, Yes}...

283. Ohfur283 Own household furniture {1, Yes}...

284. Ococh284 Own couch {1, Yes}...

285. Otab285 Own table {1, Yes}...

286. Ochar286 Own chairs {1, Yes}...

287. Obed287 Own bed {1, Yes}...

288. Odes288 Own desk {1, Yes}...

289. Tohfur289 Total house furniture {0, None}...

290. Hfurav290 Have ownership over house furniture {1, Yes}...

291. Whfpro291 What household furniture provided {1, Use a plaited mat}...

292. Profurdi292 Provided house furniture due to disaster {1, Yes}...

293. Profuby293 Who provided household furniture {0, None}...

294. Hcarhel294 Household has health card (Askeskin) {1, Yes}...

295. Hmhel295 Household member has health card {1, All household members}...

296. Hparhel296 Household participate health fund (Dana Sehat Program) {1, Yes}...

297. Hlethel297 Household utilize letter of poor (Surat Ket Tidak Mampu) {1, Yes}...

V. Community

Note: The data in this section were gathered using survey questions were drawn from well-established metrics commonly used by community scholars [6, 7, 8, 9] with prior application to countries in Southeast Asia [10] and are consistent with items recommended by disaster scholars [3, 4, 5]. More specifically, variables 310, 312, 314, and 316 are a direct replication of the community experience scale [6, 10], which was an extension of the community attachment scale [7]. Likewise, variables 311, 313, 315, and 317 are a further adaptation of the community experience scales [6, 10]. In addition, variables 318 – 323 are a direct replication of community attachment indicators [7]. Finally, variables 324 – 331 are a direct replication of sense of community indicators [8] and variables 373 – 381 are a direct replication of the evaluations of local attributes and community satisfaction indicators [9].

298. Plaberp298 Name place before eruption {1, Tunggularum}...

299. Plasam299 Place name is the same with the place of birth {1, Yes}...

300. Typla300 Type place {1, Village}...

301. Ylipp301 Years live in the previous place {1, 16}...

302. Slipp302 Years spouse live in the previous place {0, 0}...

303. Datrel303 Date force to relocate {1, 17 November 2010}...

304. Plarefg304 Place find as refugee {1, Private home-family}...

305. Tirel305 Times relocated since the eruption {1, 1 time}...

306. Ncure306 Name currently residing {1, Tunggularum}...

307. Disformv307 Distance current residence from former village {0, None}...

308. Fparc308 Feel currently part of local community {1, Yes}...

309. Fallt309 Feel things these days {1, Very unhappy}...

310. Ffitcom310 Feel fit into current community {1, Poorly}...

311. Ffitpre311 Feel fit into their previous community {1, Poorly}...

312. Hcom312 Have in common with most of people in their community {1, Nothing}...

313. Hcomp313 Have in common with most of people in their previous community {1, Nothing}...

314. Fsats314 Feel satisfied living with their community {1, Dissatisfied}...

315. Fsatspc315 Feel satisfied living with their previous community {1, Dissatisfied}...

316. Imidco316 Imagine their present community rank compare to their ideal one {1, Worst}...

317. Precoid317 Their rank their previous community compare to their ideal one {1, Worst}...

318. Perkin318 Percentage distant kin live within 50 km {1, 0 to 25%}...

319. Perspo319 Percentage distant spouse kin live within 50 km {1, 0 t0 25%}...

320. Proadl320 Proportion adult friend live in this community {1, None or very few of them}...

321. Prokno321 Proportion adult would they say known by name in the community {1, None or very few of them}...

322. Prorel322 Proportion relationships between people they know from formal setting {1, None or very few of them}...

323. Compre323 Describe likes living in the community compare to previous one {1, They would do everything possible to stay here. They don't want to go back}...

324. Getun324 You can get what you need in the community {1, Strongly agree}...

325. Comhep325 Community helps you fulfill your need {1, Strongly agree}...

326. Felmem326 You feel like a member of community {1, Strongly agree}...

327. Belcom327 You belong in the community {1, Strongly agree}...

328. Sayco328 You have a say about what goes on in the community {1, Strongly agree}...

329. Pegin329 People in the community are good in influencing each other {1, Strongly agree}...

330. Yfecon330 You feel connected to community {1, Strongly agree}...

331. Gobon331 You have a good bond with others {1, Strongly agree}...

332. Vilhed332 Village head has already been organized or appointed {1, Yes}...

333. Viof333 Village officers has been organized {1, Yes}...

334. Vilcon334 Village consultative LMD has already been organized or appointed {1, Yes}...

335. LKMD335 LKMD has already been organized {1, Yes}...

336. Hamol336 Hamlet already outlined {1, Yes}...

337. Neigh337 Neighborhood already outlined {1, Yes}...

338. Comsol338 Community solidarity units (RW) already outlined {1, Yes}...

339. Neigsol339 Neighborhood solidarity unit (RT) already outlined {1, Yes}...

340. HHo340 Head Household already outlined {1, Yes}...

341. HHmet341 Household unit meetings have been held {1, Yes}...

342. RTmet342 RT meetings have been held {1, Yes}...

343. RWmet343 RW meetings have been held {1, Yes}...

344. Vilmet344 Village meetings have been held{1, Yes}...

345. Kecmet345 Kecamatan meetings have been held {1, Yes}...

346. LMD346 LMD meetings have been held {1, Yes}...

347. LKMD347 LKMD meetings have been held {1, Yes}...

348. Volwor348 Voluntary work already organized {1, Yes}...

349. Copar349 Community participation in any of these efforts {1, Yes}...

350. Voimp350 Voluntary labor has been organized to help improve current community {1, Yes}...

351. Tpaef351 They have participated in any of these efforts {1, Yes}...

352. Relo352 Religion activities already organized {1, Yes}...

353. Parel353 Have they participated in any of these religious activities {1, Yes}...

354. Paris354 Participation in arisan {1, Yes}...

355. Taris355 Type of arisan {0, None}...

356. Aofic356 Arisan office {1, Yes}...

357. Asubn357 Arisan sub-neighborhood (RT) {1, Yes}...

358. Aneig358 Arisan neighborhood (RW) {1, Yes}...

359. Avil359 Arisan village {1, Yes}...

360. Aciv360 Arisan wives of civil servant/military {1, Yes}...

361. Apkk361 Arisan PKK {1, Yes}...

362. Amar362 Arisan market {1, Yes}...

363. Afam363 Arisan family {1, Yes}...

364. Arel364 Arisan religious group {1, Yes}...

365. Afrin365 Arisan friend {1, Yes}...

366. Aret366 Arisan retirees {1, Yes}...

367. Afarm367 Arisan farmers group {1, Yes}...

368. Ayut368 Arisan youth group {1, Yes}...

369. Amot369 Arisan motor cycle {1, Yes}...

370. Tarsm370 Times arisan meetings {0, 0}...

371. Toars371 Total amount of arisan in rupiah {0, None}...

372. Arsev372 Arisan received in total since 12 month ago {0, None}...

373. Fcbrig373 Future of this community looks bright {1, Strongly disagree}...

374. Rparf374 Resident participate in community affairs {1, Strongly disagree}...

375. Rnled375 Residents of this community receptive to new resident in leadership {1, Strongly disagree}...

376. Oinb376 Organization and groups are interested in what is the best for all {1, Strongly disagree}...

377. Pwtdo377 People in the community work together to get things done {1, Strongly disagree}...

378. Acco378 They are very active in local community improvement activities {1, Strongly disagree}...

379. Coflg379 Conflict usually doesn't take place between people in the community {1, Strongly disagree}...

380. Trup380 They trust people in this community {1, Strongly disagree}...

381. Sicul381 The people in this community have similar culture traditions and values {1, Strongly disagree}...

VI. Physical and Mental Health

Note: Some of the data in this section were gathered using survey questions that were adapted to maintain consistency with questions in either the IDHS or the IFLS [1,2]. Other data were gathered using questions that replicated well-known items that can, for example, be used to in the calculation of The Perceived Stress Scale [11], a depression scale [12], locus of control scales [13], and a loneliness scale [14]; i.e., variables 389 – 398 are a direct replication of The Perceived Stress Scale [11], variables 399 – 412 are a direct replication of a self-report depression scale [11], variables 413 – 424 are a direct replication of the locus of control scales [13], and variables 425 – 434 are a direct replication of a loneliness scale [14].

382. Pyhel382 Their physical health rate {1, Very good}...

383. Mhel383 Their mental health rate {1, Very good}...

384. Fhelp384 Find out physical or mental health problems {1, Yes}...

385. Sphel385 Specify their physical health {0, None}...

386. Smhel386 Specify their mental health {1, Insane}...

387. Comed387 Total days to consult medical healer {0, 0}...

388. Dismed388 The closest distant to medical healer {0, None}...

389. Upse389 You have been upset of something that happened unexpectedly {0, Never}...

390. Uncon390 You have felt that unable to control the important things in your life {0, Never}...

391. Fner391 Felt nervous and stress {0, Never}...

392. Fcon392 You have felt confident about the ability to handle personal problems {0, Never}...

393. Foway393 You have felt that things were going on your way {0, Never}...

394. Focop394 You have found that you could not cope with all things you had to do {0, Never}...

395. Conim395 You have been able to control imitation in your life {0, Never}...

396. Ftop396 You have felt that you were on top of things {0, Never}...

397. Ange397 You have been angered because of things that were outside your control {0, Never}...

398. Fdif398 You have felt difficulties were piling up so high that could not overcome {0, Never}...

399. Fbot399 Felt bother by things that usually not bother {0, 0 None}...

400. Fneat400 Felt like not eating, their appetite was poor {0, 0 None}...

401. Fconb401 Felt they could not shake off the blues even with help from family {0, 0 None}...

402. Trobm402 Had trouble keeping their mind on what they were doing {0, 0 None}....

403. Fdep403 Felt depress {0, 0 None}...

404. Feffor404 Felt everything they did was an effort {0, 0 None}...

405. Ffear405 Felt fearful {0, 0 None}...

406. Slepre406 Slept restlessly {0, 0 None}...

407. Tales407 Talk less than usual {0, 0 None}...

408. Flon408 Felt lonely {0, 0 None}...

409. Fsad409 Felt sad {0, 0 None}...

410. Fcog410 Felt you could not get going {0, 0 None}...

411. Tnotw411 Think that nothing worthwhile anymore {0, 0 None}...

412. Asom412 Anxious about something or someone {0, 0 None}...

413. Livdet413 I feel what happens in my life mostly determined by powerful others {1, Strongly disagree}...

414. Makpn414 When I make plan, I am almost certain to make them work {1, Strongly disagree}...

415. Getwan415 When I get what I want, it is usually because I'm lucky {1, Strongly disagree}...

416. Godab416 Although I might have good ability, I will not be given responsibility {1, Strongly disagree}...

417. Fredep417 Many friends I have depends on how nice a person I am {1, Strongly disagree}...

418. Hapen418 I have often found that what is going to happen will happen {1, Strongly disagree}...

419. Lifconp419 My life is chiefly controlled by powerful others {1, Strongly disagree}...

420. Wipla420 It's not wise to plan too far ahead because things turn out to be a matter of good or bad fortune {1, Strongly disagree}...

421. Gtwan421 When I get what I want, it's usually because I worked hard for it {1, Strongly disagree}...

422. Gtplea422 Getting what I want requires pleasing those above me {1, Strongly disagree}...

423. Beled423 Whether or not I become a leader depends on if I am lucky enough to be in the right time right place {1, Strongly disagree}...

424. Predet424 I pretty much determine what will happen in my life {1, Strongly disagree}...

425. Sotal425 There is always someone I can talk to my day-to-day problems {1, Yes!}...

426. Misfre426 I miss having a really close friend {1, Yes!}...

427. Gensen427 I experience a general sense of emptiness {1, Yes!}...

428. Plerel428 There are plenty of people I can rely on when I have problems {1, Yes!}...

429. Mispl429 I miss the pleasure of the company of others {1, Yes!}...

430. Frelim430 I find my circle of friends and acquaintances too limited {1, Yes!}...

431. Petrus431 There are many people I can trust completely {1, Yes!}...

432. Mispe432 I miss having people around {1, Yes!}...

433. Frejec433 I often feel rejected {1, Yes!}...

434. Cancal434 I can call on my friends whenever I need them {1, Yes!}...

435. Impor435 Have them imagine the poorest one to the richest which step they place themselves today {1, Poorest}...

436. Imsteb436 Have imagine on which step were you before eruption {1, Poorest}...

437. Imfiv437 Have imagine on which step do you expect to find yourself five years from now {1, Poorest}...

VII. Household Members

Note: The data in this section were gathered using a household member table that was adapted to maintain consistency with the tables in the IDHS and the IFLS [1, 2] that are used to document all members of a household.

438. H1me438	HH member name	{0, None}...
439. H1ma439	HH member age	None
440. H1mar440	HH member marital status	{1, Not married}...
441. H1rel441	HH member religion	{1, Islam}...
442. H1eth442	HH member ethnicity	{1, Javanese}...
443. H1ed443	HH member education	{1, Primary school}...
444. H2me444	H2 member name	{0, None}...
445. H2ma445	H2 member age {0, 0}...	
446. H2mar446	H2 member marital status	{1, Not married}...
447. H2rel447	H2 member religion	{1, Islam}...
448. H2eth448	H2 member ethnicity	{1, Javanese}...
449. H2ed449	H2 member education	{0, Not yet go to school}...
450. H3me450	H3 member name	{0, None}...
451. H3ma451	H3 member age {0, 0}...	
452. H3mar452	H3 member marital status	{1, Not married}...
453. H3rel453	H3 member religion	{1, Islam}...
454. H3eth454	H3 member ethnicity	{1, Javanese}...
455. H3ed455	H3 member education	{0, Not yet go to school}...
456. H4me456	H4 member name	{0, None}...
457. H4ma457	H4 member age {0, 0}...	
458. H4mar458	H4 member marital status	{1, Not married}...
459. H4rel459	H4 member religion	{1, Islam}...
460. H4eth460	H4 member ethnicity	{1, Javanese}...

461. H4ed461 H4 member education {0, Not yet go to school}...

462. H5me462 H5 member name {0, None}...

463. H5ma463 H5 member age {0, 0}...

464. H5mar464 H5 member marital status {1, Not married}...

465. H5rel465 H5 member religion {1, Islam}...

466. H5eth466 H5 member ethnicity {1, Javanese}...

467. H5ed467 H5 member education {0, Not yet go to school}...

468. H6me468 H6 member name {0, None}...

469. H6ma469 H6 member age {0, 0}...

470. H6mar470 H6 member marital status {1, Not married}...

471. H6rel471 H6 member religion {1, Islam}...

472. H6eth472 H6 member ethnicity {1, Javanese}...

473. H6ed473 H6 member education {0, Not yet go to school}...

474. H7me474 H7 member name {0, None}...

475. H7ma475 H7 member age {0, 0}...

476. H7mar476 H7 member marital status {1, Not married}...

477. H7rel477 H7 member religion {1, Islam}...

478. H7eth478 H7 member ethnicity {1, Javanese}...

479. H7ed479 H7 member education {0, Not yet go to school}...

480. H8me480 H8 member name {0, None}...

481. H8ma481 H8 member age {0, 0}...

482. H8mar482 H8 member marital status {1, Not married}...

483. H8rel483 H8 member religion {1, Islam}...

484. H8eth484	H8 member ethnicity	{1, Javanese}...
485. H8ed485	H8 member education	{0, Not yet go to school}...

References

[1] Statistics Indonesia and Macro International. 2008. Indonesia Demographic and Health Survey 2007. Calverton, Maryland, USA: BPS and Macro International.

[2] Strauss, J., F. Witoelar, B. Sikoki and A.M. Wattie. "The Fourth Wave of the Indonesian Family Life Survey (IFLS4): Overview and Field Report". April 2009. WR-675/1-NIA/NICHD.

[3] Cope, Michael R., Tim Slack, Troy C. Blanchard, Matthew R. Lee, and Jorden E. Jackson[1]. 2020. “The Louisiana Community Oil Spill Survey (COSS) Dataset.” *Data in Brief* 74 (February):124-132

[4] Lindell, Michael K., and Carla S. Prater. "Assessing community impacts of natural disasters." *Natural hazards review* 4.4 (2003): 176-185.

[5] Norris, Fran H., et al. "Community resilience as a metaphor, theory, set of capacities, and strategy for disaster readiness." *American journal of community psychology* 41.1-2 (2008): 127-150.

[6] Kasarda, John D., and Morris Janowitz. "Community attachment in mass society." *American sociological review* (1974): 328-339.

[7] Brown, Ralph B., Xiaohe Xu, Melissa A. Barfield, and Brayden G. King. "Community experience and the conceptual distinctness of rural community attachment and satisfaction." Research in Community Sociology 10 (2000): 421-446.

[8] Peterson, N. Andrew, Paul W. Speer, and David W. McMillan. "Validation of a brief sense of community scale: Confirmation of the principal theory of sense of community." *Journal of community psychology* 36, no. 1 (2008): 61-73.

[9] Goudy, Willis J. "Evaluations of local attributes and community satisfaction in small towns." *Rural sociology* 42, no. 3 (1977): 371.

[10] Muir, Jonathan A., David B. Braudt, Jeffrey Swindle, Jeremy Flaherty, and Ralph B. Brown. "Cultural antecedents to community: An evaluation of community experience in the United States, Thailand, and Vietnam." City & community 17, no. 2 (2018): 485-503.

[11] Cohen, S., Kamarck, T., and Mermelstein, R. (1983). A global measure of perceived stress. Journal of Health and Social Behavior, 24, 386-396.

[12] Radloff, Lenore S. 1977. “The CES-D Scale: A Self-Report Depression Scale for Research in a General Population.” Applied Psychology Measurement 1:385–401

[13] Levenson, Hanna. "Activism and powerful others: Distinctions within the concept of internal-external control." *Journal of personality assessment* 38.4 (1974): 377-383.

[14] De Jong-Gierveld, Jenny, and Frans Kamphuls. "The development of a Rasch-type loneliness scale." *Applied psychological measurement* 9.3 (1985): 289-299.
